# In a sea of microbes, eddy events trigger diatom export in the Sargasso Sea

**DOI:** 10.1093/ismeco/ycaf083

**Published:** 2025-05-19

**Authors:** Marc Alec Fontánez Ortiz, Francesca De Martini, Susanne Neuer

**Affiliations:** School of Life Sciences, Arizona State University, Tempe, AZ 85287, United States; School of Earth and Space Exploration, Arizona State University, Tempe, AZ 85287, United States; Center for Fundamental and Applied Microbiomics, The Biodesign Institute, Arizona State University, Tempe, AZ 85287, United States; Life Science Department, Mesa Community College, Mesa, AZ 85202, United States; School of Life Sciences, Arizona State University, Tempe, AZ 85287, United States; Center for Fundamental and Applied Microbiomics, The Biodesign Institute, Arizona State University, Tempe, AZ 85287, United States; School of Ocean Futures, Arizona State University, Tempe, AZ 85287, United States

**Keywords:** marine microbial communities, sinking particles, Sargasso Sea, mesoscale eddies, diatoms

## Abstract

Sinking particles are important conduits of organic carbon from the euphotic zone to the deep ocean, but their origin and community composition are still a matter of investigation. Events in the northwestern Sargasso Sea, such as winter convective mixing, summer stratification, and mesoscale eddies, affect the vertical and temporal composition and abundance of pelagic and particle-attached microorganisms. We sampled the euphotic zone and collected sinking particles using shallow traps near the Bermuda Atlantic Time-series Study site during the spring and summer of 2012 to assess eddy-driven impact on microbial communities. In the spring, we sampled a cyclonic eddy, while in the summer, we targeted both the center and edge of an anticyclonic eddy. Prokaryotic and photoautotrophic (plastid and cyanobacteria) communities were analyzed using V4–V5 amplicons of the 16S rRNA gene. Community and clustering analysis of prokaryotes revealed a clear separation between seawater and particles samples. However, the same was not observed for photoautotrophs. Indicator species analysis showed that small phytoplankton taxa dominated particle communities. Interestingly, differential abundance analyses revealed that the large centric diatom, *Rhizosolenia*, generally rare in the oligotrophic Sargasso Sea, was enriched in the photoautotrophic communities of sinking particles collected in the center of the anticyclonic eddy with unusual upwelling due to eddy–wind interactions. We hypothesize that the steady contribution of small-celled phytoplankton to particle flux is punctuated by pulses of production and flux of larger-sized phytoplankton in response to episodic eddy upwelling events and can lead to higher export of particulate organic matter during the summer.

## Introduction

Oligotrophic gyres are the ocean’s desert and comprise 40% of the Earth’s surface [1]. They are characterized by low productivity, low phytoplankton biomass, and small-sized phytoplankton (generally <5 μm) relative to eutrophic areas [2, 3]. The Sargasso Sea experiences annual deep convective mixing during the winter [4, 5], deepening the mixed layer with depths ranging from 100 to 400 m [6]. This mixing with nutrient-rich water from below [7] results in higher phytoplankton biomass and “new” primary production, leading to the winter–spring bloom [3, 6, 8].

Mesoscale eddies are a prominent physical forcing mechanism in the Sargasso Sea [9, 10], and can introduce spatial heterogeneity and variability across scales of 10s to 100s of kilometers [11]. These eddies impact primary production most significantly at their core, but their edges and interactions between adjacent eddies can have different effects on the plankton community [11]. Young eddies (<4 months) usually show a stronger biological response than their older counterparts [11, 12]. In fact, the influence of these eddies on primary productivity surpasses the seasonality of mixing and stratification previously mentioned [8, 13]. Consequently, understanding eddy behavior is important when evaluating the seasonality of the plankton community and its impact on export production.

Through photosynthesis, phytoplankton fix dissolved inorganic carbon into organic matter, playing a key role in the global carbon cycle and in carbon export [14]. Organic matter is exported mainly as particulate organic carbon by the gravitational sinking of particles [15, 16]. The export and sinking velocity of the particles out of the euphotic zone (EZ) depend on their composition, size, and density, which is also influenced by transparent exopolymeric particles (TEP) that make up the matrix of many aggregates [17–19]. The collision of marine aggregates with biogenic and lithogenic ballasting minerals can also enhance the particle’s density. Zooplankton play an important role as they either fragment and/or “repackage” marine particles [20], producing fecal pellets that can sink or interact with other aggregates. Viruses can influence microbial dynamics and particle aggregation, for example, by enhancing the production of TEP as has been found in blooms of coccolithophores [21].

Heterotrophic bacteria are contained in and colonize marine particles [22], which enhance aggregation by the production of TEP, thereby increasing stickiness [18]. Hence, marine aggregates also serve as microhabitats for diverse microbial communities whose abundance is 3–5 log-fold higher than surrounding seawater [23]. Bacteria in marine aggregates can promote fast particulate organic matter turnover [24, 25] and therefore play an essential role in the degradation of aggregates as they are exported to depth [26]. The carbon export can be measured using surface-tethered Particle Interceptor Traps (PITs) [27], which allows the collection of sinking particles [28] and quantification of POC export—an approach adapted in this study.

Amacher *et al.* [29] carried out the first DNA-based investigation of cyanobacteria and protist communities collected by particle traps in the Sargasso Sea, comparing their occurrence in the EZ and sinking particles recovered at 150 m depth through monthly sampling over two years. They found small sized phototrophs such as prasinophytes in the particle traps, and greater eukaryotic than cyanobacterial richness after deep mixing events due to increased storm activity and cyclonic eddy events. Interestingly, they found that the coccoid cyanobacteria *Synechococcus* were overrepresented in the particle traps compared to the seawater, while the cyanobacteria *Prochlorococcus* were underrepresented in the trap material. Later studies investigating different particle types collected with PITs at BATS identified prokaryotic taxa associated with the gut microbiome of zooplankton to be part of the core microbiome of sinking particles [30].

In this study, we investigate the importance of mesoscale eddies on the community composition of prokaryotes and photoautotrophs (plastid and cyanobacteria) in the EZ and sinking particles collected below the EZ in the Sargasso Sea. This study adds to earlier work [31, 32] that investigated the influence of mesoscale features on the composition, production, and export of the plankton community in this oligotrophic ecosystem.

## Materials and methods

### Sample collection and processing

The investigation was carried out on the *R/V* Atlantic Explorer in March and July 2012 in the northwestern Sargasso Sea, targeting mesoscale eddies (Supplementary Fig. 1), as described elsewhere [31–33]. In March 2012, the center of a 6-month-old cyclonic eddy (C2) and the Bermuda Atlantic Time-series Study (BATS) site were sampled. In July 2012, samples were collected from the center and the edge of a <1-month-old anticyclonic eddy (AC2, ACe2) as well as from BATS, influenced by the edge of another cyclone. A 3-day deployment of formalin-poisoned surface-tethered Particle Interceptor Traps was carried out at 150 m depth at the four sites according to BATS standard protocols (http://bats.bios.edu/) to study the microbial community associated with sinking particles (Table 1). The water column of all stations was sampled twice, at deployment and 48 h later at 20 m and the deep chlorophyll maximum that ranged from 80 to 100 m depth. Processing of samples from replicate trap tubes was done according to De Martini *et al.* [32].

**Table 1:** Cruise numbers and season/year, station names, dates of deployment and recovery of the PITs, and coordinates of trap deployment.

Cruise	Season	Station	PITs deployment	PITs recovery	Latitude	Longitude
AE1206	Spring 2012	B3	15-Mar	17-Mar	32° 50′N	63° 30′W
		C2	19-Mar	21-Mar	31° 40′N	64° 10′W
AE1219	Summer 2012	AC2	20-Jul	22-Jul	33° 30′N	64° 27′W
		Ace2	24-Jul	26-Jul	32° 22′N	64° 22′W
		B4	28-Jul	30-Jul	31° 40′N	64° 10′W

BATS spring (B3) and summer (B4); anticyclonic eddy center (AC2) and edge (ACe2).

### DNA extraction and 16S rRNA gene sequencing

The DNA of EZ and trap samples was extracted using a QIAGEN DNeasy Blood and Tissue Kit [32]. 16S rRNA gene amplicon libraries were prepared and sequenced using an Illumina MiSeq platform (reagent kit v3; 2 × 300 bp paired-end; Illumina Inc.) by the Dalhousie University Integrated Microbiome Resource (IMR). The universal V4–V5 primer set 515F-Y (5′-GTGYCAGCMGCCGCGGTAA-3′) [34] and 926-R (5′-CCGYCAATTYMTTTRAGTTT-3′) [34, 35] were employed. The demultiplexed reads received from IMR were analyzed using QIIME2 (v2024.2) [36]. Sequences were classified using the SILVA (v 138.1) database [37] and the PR2 (v 15.0) database [38] for the photoautotrophs. PR2 includes plastidial 16S rRNA gene reference sequences from the PhytoREF database [39] as of April 2021. Saturation curves (Supplementary Figs 2–3) were used to validate the effects of rarefaction depth on diversity metrics (Supplementary Tables 1–2). Additional details on amplicon sequence variant (ASV) preparation, diversity analysis, and our rationale on rarefaction and normalization are found in the Supplementary Material.

### Statistical analysis

Statistical modeling was performed within R statistical software (v4.3.2) by creating an R object of the rarefied libraries using the *phyloseq* package (v1.46.0) [40]. For statistical analysis that required randomization, eight random values were repeatedly used as seed for reproducibility. The rarefied ASV tables in the *phyloseq* objects were used to prepare a non-metric multidimensional scaling (NMDS) ordination based on Bray–Curtis dissimilarity distances. The differences associated with sample types (seawater and trap material), season (spring and summer) as well as depth (20 m, DMC, and 150 m) were visualized.

A pairwise permutational multivariate analysis (PERMANOVA) [41] was performed within the *metaMDS* and *adonis* functions from the *vegan* (v2.6–4) package [42] using the same factors from the NMDS and separately comparing seawater and bulk particle trap samples. To decrease the probability of falsely rejecting the null hypothesis due to multiple comparisons, the false discovery rate (*FDR*) method [43] was implemented using the base R function, *p.adjust*.

Heatmaps of the top 45 most abundant ASVs were generated using the *phyloseq* object. We first applied a log(x + 1) transformation before computing a distance matrix using Bray-Curtis dissimilarities (see Supplementary Text for more details). Heatmaps were generated using the *pheatmap* (v1.012) [44] package. The ASVs (i.e. rows) were clustered based on Bray-Curtis dissimilarities using the default Unweighted Pair Group Method with Arithmetic Mean (UPGMA). The hierarchical clustering analysis of samples (i.e. column) was pre-computed and performed on the same log(x + 1)-transformed rarefied tables used for the heatmap. We applied UPGMA along with a bootstrapping approach provided by the *pvclust* (v2.2-0) package to determine statistically significant clusters and branches based on approximately unbiased (AU) *P*-values (1000 iterations; 5% significance level).

To establish the strength of ASV associations with a priori groups (seawater and particulate trap material), we calculated the indicator value (IndVal) using the *multipatt* function from the *indicspecies* package (v1.7.15) [45] on the rarefied ASV tables in *phyloseq*. This test identifies the *fidelity* of the ASVs, and *specificity* of the ASVs to either or both seawater and particulate trap material. To report the group-wise IndVal, the *p-value* was adjusted for multiple testing issues, as advised by De Cáceres *et al.* [45], only ASVs with an adjusted *P* ≤ .05 were selected using the Benjamini–Hochberg procedure [46]. Further details are available in the Supplementary Text.

Lastly, the *DESeq2* package (v 1.42.0) [47] facilitated a differential abundance analysis of ASVs to pinpoint the community variance between seawater and particle trap material across seasons. Before the analysis, the ASV table in the *phyloseq* object underwent transformation by pruning samples that were not part of the groups of interest (i.e. excluding summer samples during spring sample analysis and vice versa) to ensure the correct within-season calculation of the geometric means. After running the differential abundance analysis, hypothesis testing was done by using the Wald test for a two-group comparison [48], and the *P-value* was adjusted using the Benjamini–Hochberg procedure [46]. The ASVs with a significant (*P* ≤ .05) log_2_-fold change (L2FC) between seawater and particle trap material from each season were plotted. These plots were then color-coded with the average percent relative abundance calculated from the normalized mean counts of the samples relative to the targeted season comparison. While the L2FC indicates environmental preference, the mean abundance shows how dominant the ASVs are within each environment.

## Results

### Environmental conditions

During our study, we targeted cold-core (cyclonic) and warm-core (anticyclonic) mesoscale eddies in the vicinity of BATS during early spring and mid-summer 2012 [31]. In March 2012, the 6-month-old C2 northeast of BATS showed a cold-core (19.5°C), low-nutrient inventories, and had a deeper mixed layer depth (~150 m) compared to BATS (20.5°C; MLD 65–135 m), which was not influenced by an eddy. The slightly colder temperatures at the core, in comparison to BATS, are indicative of an upwelling event [11, 49]. However, the lower nutrient inventory in the cyclone suggests it was either a decaying feature or had minimal mixing from outside of the eddy [12, 50]. In July 2012, we sampled the center and edge of a less than a one-month-old anticyclonic eddy (AC2 and ACe2, respectively) northwest of BATS that exhibited upwelling [31, 32], attributed to eddy/wind interactions [51]. All summer stations presented a well-stratified water column with temperatures ranging from 28°C at the surface to 20°C at the base of the EZ and shallow mixed layers, with the shallowest depth at BATS (15–22 m), followed by the center (18–24 m) and edge (25–34 m) of the anticyclone. BATS was influenced by the edge of a young cyclone located southeast of the site during that time.

### Prokaryotic community analysis

**Figure 1:**
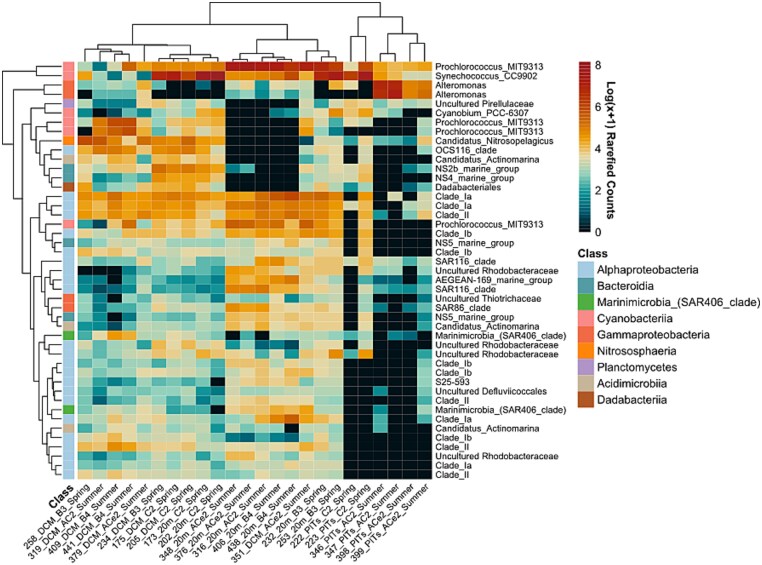
Heatmap of the top 45 most abundant prokaryotic ASVs, represented as log(x + 1)-transformed rarefied counts. Rows correspond to ASVs (with the corresponding genus shown on the right side), and columns represent samples. The top dendrogram illustrates sample clustering, while the left side dendrogram depicts ASV clustering, both based on Bray–Curtis dissimilarity of the transformed counts and visualized using UPGMA. A class-level color-code bar shows class-level classification of each ASV.

Alpha-diversity of prokaryotes (Supplementary Fig. 4) showed lower richness and evenness in spring seawater communities compared to summer, while summer particles trap libraries presented lower Shannon diversity but higher phylogenetic richness (Faith’s PD), likely due to additional ASVs such as *Alteromonas* (Fig. 1). The relative abundance of the rarefied dataset (Supplementary Fig. 5) showed that *Alphaproteobacteria* made up 11% of the relative abundance in particle libraries and 32% in ambient seawater, while in the summer the relative abundance was 7% in particle libraries and 39% in ambient seawater. In contrast, *Gammaproteobacteria* were predominantly found in particle libraries (spring: 19%; summer: 64%), while their relative contribution to the water column microbial community was lower in both seasons (spring: 7%; summer: 9%). Bacteria of the class *Bacteroidia* were more abundant in the particle libraries (spring: 27%; summer: 13%) than in the seawater (spring: 11%; summer: 1%), where 20% of relative contribution from *Bacteroidia* was attributed to one replicate of the spring C2 (sample 222). *Cyanobacteria* generally had higher relative contributions in the ambient seawater (spring: 34%; summer: 27%), but they also followed suit in the spring particle libraries (spring: 32%; summer: 4%). Additionally, one *Synechococcus* ASV was overrepresented in spring seawater, while one *Prochlorococcus* ASV dominated the summer seawater libraries. Moreover, a strong co-occurrence of *Prochlorococcus* and *Synechococcus* ASVs in the water column was found during both seasons at BATS at 20 m depth. SAR11 ASVs pertaining to the clades Ia and Ib were also important contributors to the seawater community, and clade II to a lesser extent. The particle communities collected in the anticyclonic eddy were dominated by ASVs of *Alteromonas* at the core and edge of the mesoscale feature, while those collected in the eddy C2 (mainly sample 223) were dominated by ASVs of *Synechococcus* followed by *Prochlorococcus*.

The communities of prokaryotes showed overall compositional differences between the seawater and particulate trap libraries as revealed by the NMDS (stress = 0.121) and shown in Supplementary Fig. 6. This was assessed via PERMANOVA (Supplementary Table 3), revealing that location (B3, C2, AC2, ACe2, and B4) significantly explains 61.2% of the community variation across both seasons (FDR-corrected *P* ≤ .05), likely driven by particle libraries, which explained 81.6% of the community for both seasons and 89.6% for the summer variation in the trap libraries. Particle and seawater communities grouped in two separate major clusters with branching structure influenced by season as revealed by the bootstrapping at 95% significance level performed for the UPGMA dendrogram of prokaryotes (Supplementary Fig. 7).

**Figure 2:**
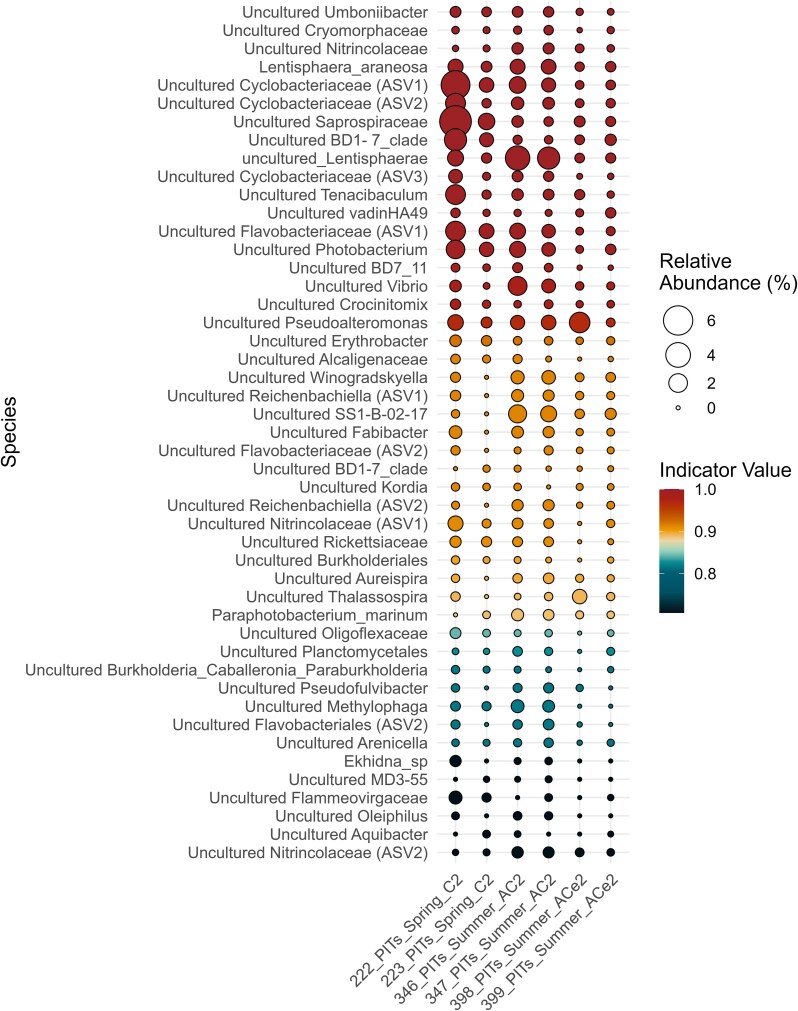
Balloon plot depicting relative abundance of significant prokaryotic indicator ASVs (*P* < .05) for the trap material collected in spring and summer. The size of the bubbles depicts the relative abundance for each sample and the color shows IndVal range. Only significant ASVs with IndVal ≥0.8 were considered as indicators.

After correcting for multiple comparisons to identify the list of indicators in each sample type (Fig. 2), we found 47 ASVs to be part of the core microbiome of the particle communities, and 15 ASVs in the seawater communities in both seasons. Indicators for seawater were ASVs predominantly within *Proteobacteria*, such as the Alphaproteobacteria order *Rickettsiales*, the Gammaproteobacteria order *Pseudomonadales*, and the Alphaproteobacteria SAR11 clade. In contrast, the indicator ASVs for the trap samples were dominated by *Bacteroidota*, *Proteobacteria*, and, to a lesser extent, *Verrucomicrobiota*. Three of the four indicator ASVs in the bulk particle sample with the highest IndVal possible were from the Gammaproteobacteria order *Pseudomonadales*, one of which was *Umboniibacter.* In addition, proteobacterial ASVs that have been previously linked to sinking particles [30, 52] were also present as indicator species, such as *Vibrio*, *Pseudoalteromonas*, and *Erythrobacter* with high IndVal (≥ 0.8; FDR-corrected *P* ≤ .05). Alphaproteobacterial ASVs mainly dominated as indicators for the seawater libraries of both seasons (Supplementary Table 5).

**Figure 3:**
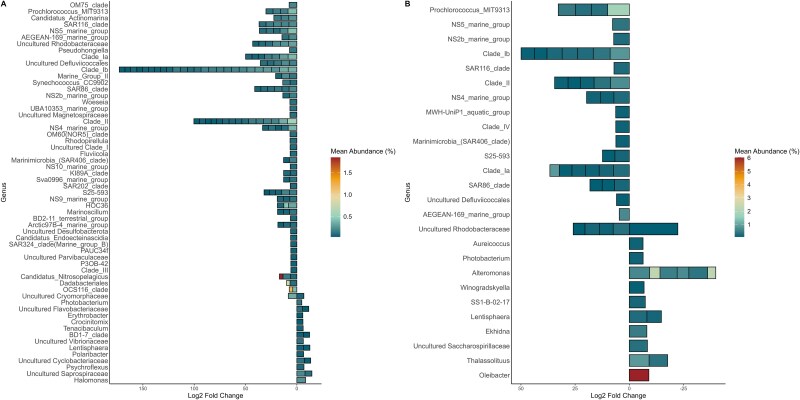
Difference in abundance of prokaryotes expressed as significant (FDR, *P* < .05) fold change between normalized taxonomic counts in seawater (positive values) and trap material (negative values) from the (A) spring and (B) summer seasons. The color represents the average abundance of mean normalized counts in all samples.

ASVs identified as indicators of the particle trap material in this study, such as those from the genera *Reinchenbachiella*, *Photobacterium*, *Lentisphaera*, *Erythrobacter,* and *Vibrio*, were also significantly differentially abundant (FDR-corrected *P* ≤ .05) in the trap material from the spring (Fig. 3A). Indicators identified for seawater also drove community differences in the spring samples, like *Rhodobacteraceae*, SAR11 clade, and *Dadabacteriales*. Indicator ASVs for particle trap material were less present in the differential abundance analysis for summer (Fig. 3B), but the ones present were uncultured *Rhodobacteraceae*, *Winogradskyella* and *Lenthisphaerae*. *Prochlorococcus* and SAR11 primarily drove the differential abundance in seawater, and *Synechococcus* was also differentially abundant in the seawater during both seasons, but not differently abundant in the bulk particle material. Notwithstanding, strong presence of one *Synechococcus* ASV was found in the spring eddy C2 particle communities, similar to observations from Amacher *et al.* [29], indicating community similarity of the spring bulk particle traps to the surrounding seawater.

### Photoautotrophic community analysis

Alpha-diversity of photoautotrophs (Supplementary Fig. 8) showed higher observed richness (ASVs) in summer seawater but reduced in summer particle samples, and consistently higher phylogenetic richness (Faith’s PD) in summer libraries. Evenness and Shannon diversity increased with depth in both seasons, with the summer particle libraries having the highest values. The relative abundance (Supplementary Fig. 9) showed that *Coscinodiscophyceae* (centric diatoms) made important contributions to the anticyclonic eddy trap samples, likely explaining the higher evenness and phylogenetic diversity seen in alpha-diversity metrics. The spring trap communities presented a similar class-level structure to the surrounding seawater, but with more contribution from *Palmophylophyceae* (chlorophytes). One ASV from the centric diatom *Rhizosolenia setigera* (now within the *Coscinodiscophyceae* [53]) represented 14% of the combined relative abundance within summer AC2 and Ace2 trap samples, but <1% in the water column of both seasons and the spring particle community.

**Figure 4:**
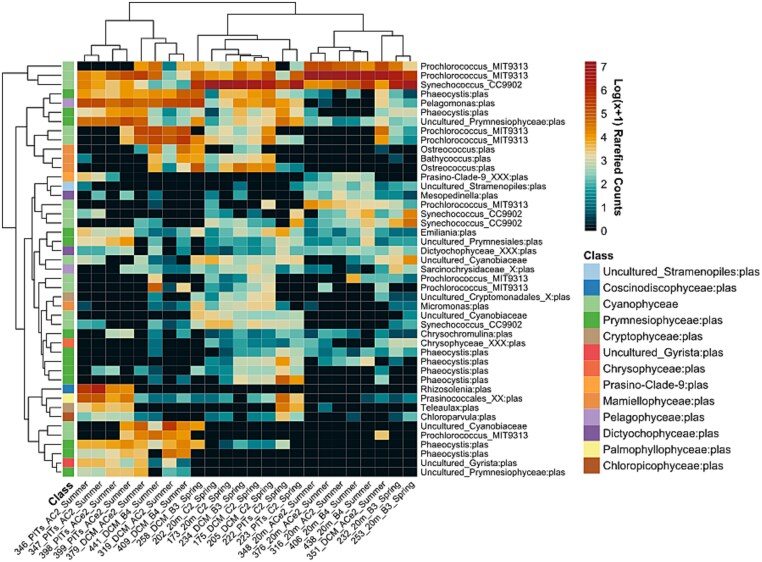
Heatmap of the top 45 most abundant photoautotrophic (plastids and cyanobacteria) ASVs, represented as log(x + 1)-transformed rarefied counts. Rows correspond to ASVs (with the corresponding genus shown on the right side), and columns represent samples. The top dendrogram illustrates sample clustering, while the left side dendrogram depicts ASV clustering, both based on Bray–Curtis dissimilarity of the transformed counts and visualized using UPGMA. A class-level color-code bar shows class-level classification of each ASV.

*Rhizosolenia* was the only diatom in the top 45 most abundant ASVs, dominating the particle libraries of the anticyclonic eddy (Fig. 4). The *Mamiellophyceae* mostly made important contributions in the DCM, particularly ASVs of *Ostreococcus* and *Bathycoccus*. However, an ASV of the small-celled phytoplankton within the *Pelagophyceae*, *Pelagomonas*, was overrepresented in both the DCM and particle trap samples of the anticyclonic eddy. Lastly, *Cyanophyceae* was the most represented class in the libraries, where *Prochlorococcus* had 8 ASVs and *Synechococcus* had 4 ASVs in the top 45. However, *Prochlorococcus* ASVs were less abundant in the spring (seawater: 17%, trap material: 5%) and more abundant in the summer (seawater: 62%, trap material: 7%) but consistently overrepresented in the seawater. The inverse trend was exhibited by *Synechococcus* ASVs, being abundantly present in the spring (seawater: 51%, trap material: 31%) and less abundant in the summer (seawater: 7%, trap material: 4%).

**Figure 5:**
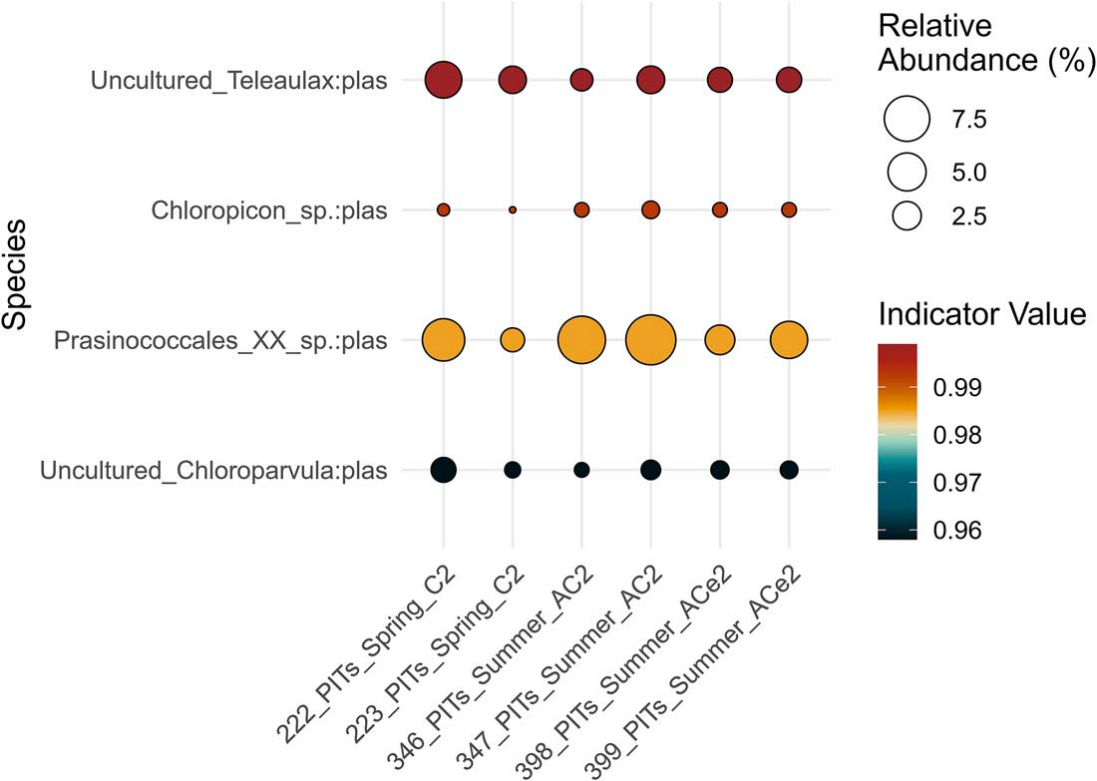
Balloon plot depicting relative abundance of significant photoautotrophic indicator ASVs (*P* < .05) for the trap material collected in spring and summer. The size of the bubbles depicts the relative percent abundance for each sample and the color shows IndVal range. Only significant ASVs with IndVal ≥0.8 were considered as indicators.

Photoautotroph communities were similar between the two sample types, as indicated by the NMDS (stress = 0.122) with overlapping 95% confidence ellipses (Supplementary Fig. 10). Assessment of the overall community structure difference (PERMANOVA; Supplementary Table 4) revealed that sample type (seawater versus bulk particle samples) significantly explained 67.5% of the community variation observed in Bray–Curtis dissimilarity of the rarefied table across both seasons (FDR-corrected *P* ≤ .05). However, this distinction was less pronounced during the spring season, explaining only 36.7% of the variation. The spring particle libraries collected in the C2 clustered together but alongside the seawater communities as indicated by the bootstrapping at 95% confidence level on the UPGMA dendrogram (Supplementary Fig. 11). In summer, particles communities significantly clustered together, but apart from the DCM and 20 m communities that were part of two other clusters (except for one DCM of the anticyclone edge). Additionally, one *Prochlorococcus* ASV was a significant indicator in the seawater libraries (Supplementary Table 6). We also found 4 other ASVs to be indicators of particle libraries (Fig. 5): the cryptophyte *Teleaulax* and three members of prasinophytes, *Prasinococcales*, *Chloropicon*, and *Chloroparvula*, the latter two being part of the relatively novel class *Chloropicophyceae* [54].

**Figure 6:**
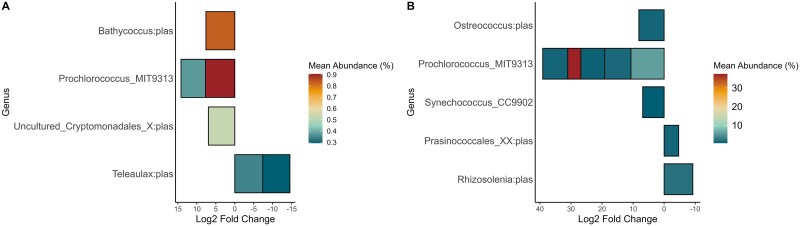
Difference in abundance of photoautotrophs expressed as significant (FDR, *P* < .05) fold change between normalized taxonomic counts in seawater (positive values) and trap material (negative values) from the (A) spring and (B) summer seasons. The color represents the average abundance of mean normalized counts in all samples.

Within the spring EZ community, an ASV of *Prochlorococcus* had the highest L2FC value and mean abundance within the season (Fig. 6A), followed by *Bathycoccus* and an uncultured cryptophyte of the *Cryptomonadales* order. Two *Teleaulax* ASVs were the only differently abundant taxa in the spring trap libraries, one of which was also identified as an indicator for the same libraries. In summer, *Prochlorococcus* was represented by five ASVs, one of which had the highest L2FC in the analysis, while another had the highest abundance, but the lowest L2FC (Fig. 6B). The latter indicates a presence in spring particle libraries, corroborated by the heatmap (Fig. 4). ASVs from *Ostreococcus* and *Synechococcus* were also differently abundant in the seawater. Lastly, the differential abundance analysis revealed that the centric diatom, *Rhizosolenia*, had an ASV significantly present (FDR-corrected *P* ≤ .05) in the summer particle community, having the highest mean abundance and L2FC within the particles. The strong presence of *Rhizosolenia* was contributed mostly by the libraries collected in the center of the anticyclonic eddy (Fig. 4). Interestingly, the *R. setigera* ASV was also identified as an indicator of the summer particle trap material, agreeing with their relative abundance contribution and L2FC results. An ASV from *Prasinococcales* was also overrepresented in the summer trap libraries and identified as an indicator for both seasons.

## Discussion

### An unusual anticyclonic upwelling event

The presence of the large centric diatom *R. setigera* in the particle traps deployed in the center of the warm-core eddy, while being nearly absent in the EZ, indicates the sedimentation of a bloom event that happened before the arrival at the site. Our 16S rRNA gene amplicon analysis is further corroborated by the pigment analyses for the same sampling stations conducted by Cotti-Rausch *et al.* [31]. They determined the phytoplankton composition and biomass for the upper 100 m depth, and generally found little diatom presence in the EZ during both seasons. While surface-tethered Particle Interceptor Traps are often biased toward smaller or slower-sinking particles (e.g. ~100 m d^−1^), they can also intercept more rapidly sinking particles, regardless of size, during episodic flux events [55, 56]. The detection of the *Rhizosolenia* ASVs—a diatom genus often associated with episodic export events [57]—in the shallow particle traps in summer despite their minimal representation in concurrently collected water column samples [31, 33], suggests that this was a rapid sedimentation event. While anticyclones are not characterized by upwelling in their center [11], the eddy/wind interactions proposed to affect this feature [31, 32] and the increased particle flux at the eddy center (21.9 POC mg C m^−2^ d^−1^) compared to the eddy edge (7.9 POC mg C m^−2^ d^−1^), and BATS (5.3 POC mg C m^−2^ d^−1^) as reported by De Martini and colleagues [32], points to the contributions of the diatoms to the POC export at this site.

### Prokaryotic community structure of seawater vs bulk particle libraries

The communities of the sinking particles collected in this study were overrepresented by *Gammaproteobacteria*, consistent with other studies [28, 30, 52] (Fig. 1; Supplementary Fig. 5). However, the mechanisms that lead to their enrichment in the marine particles may vary. For example, Raina *et al.* [58] used in situ chemotaxis assays to link motility and carbon substrate preference. They found that members of *Pseudoalteromonas* and *Vibrio* were generalist, motile taxa significantly enriched in most of the phytoplankton-derived dissolved organic matter they studied, including those produced by *Synechococcus*. This motility increases particle encounter rates on the scale of minutes to hours [59], therefore highlighting an important behavior in the patchy nutrient environments of the oligotrophic Sargasso Sea. In addition to degradation, heterotrophic bacteria have been demonstrated to enhance TEP formation and aggregation in separate xenic culture of *Prochlorococcus* and *Synechococcus* [18]. Bacteria-mediated aggregation of phytoplankton is hypothesized to be influenced by specific phytoplankton-bacteria interactions. For example, co-culture studies demonstrated that aggregation of *Minutocellos polymorphus* was enhanced by the presence of *Marinobacter adherence* HP15 but not of *Pseudoalteromonas carrageenovora* or *Vibrio thalassae* [60].

The indicator species analysis (Fig. 2) also revealed copiotrophic microbes commonly known to colonize particles, like *Pseudoalteromonas* and *Vibrio* [30, 52]. Interestingly, the ASV with the highest IndVal was *Umboniibacter*, previously isolated from tissues of marine mollusks [61], and also reported as an indicator by Cruz *et al.* [30]; however, it was one of the less abundant indicators within this study. Furthermore, one *Halomonas* ASV was significantly differently abundant in spring particle traps relative to the seawater (Fig. 3A), while ASVs from *Oleibacter*, *Alteromonas*, and other important hydrocarbonoclastic microbes were indicators in the summer particle traps in spring (Fig. 3B). The presence of *Oleibacter* in marine particles has been previously found in oligotrophic regions of the equatorial Pacific [62], where *Chaetoceros*, *Emiliania*, and *Thalassiosira* were presumably the dominant primary producers. In this study, *Oleibacter* could be associated with one of the generalist phytoplankton identified for the EZ and marine particles from both seasons, *Emiliania* (Supplementary Table 5) or to *Chaetoceros*, found to be an indicator for the particle trap libraries in summer.

The relative abundance of *Photobacterium* and *Tenacibaculum* was highest at the particle samples collected in the spring cyclone, conversely, lowest in particles collected at the edge of the summer anticyclone. Only *Pseudoalteromonas* occurred in consistently high abundance within particles of all eddy-impacted stations. The indicator and differential abundance analysis of the prokaryotes (Figs 2–3) revealed that *Photobacterium* were indicators of trap sample libraries and differently abundant in both seasons. Similarly, *Tenacibaculum* was an indicator for the bulk trap samples, but only differently abundant in the spring. *Photobacterium* is a generalist chemotactic bacterium [58] involved in nitrogen cycling [63], and *Tenacibaculum* is an important marine fish pathogen [64] usually categorized as a surface-associated microbe [65]. Size-fractionated studies from the Mediterranean Sea revealed that *Tenacibaculum* was an indicator of larger size fractions (>0.3 μm) in a prymnesiophyte-dominated region, with episodic dominance of diatoms [66]. Interestingly, *Flavobacteriaceae* were indicators of particles from both seasons (Fig. 2), but had greater contribution to particles from the spring (Fig. 3A) while *Rhodobacteraceae* ASVs had dual contributions in the seawater and particles from spring with the highest L2FC in the summer particle trap samples (Fig. 3B). Both *Flavobacteriaceae* and *Rhodobacteraceae* have been described as primary colonizers of particles from the EZ [20, 67].

The differential abundance analysis highlighted distinct patterns of microbial transport and enrichment into the bulk particle samples. In the spring (Fig. 3A), the majority of ASVs exhibited positive L2FC values, indicating that these were more abundant in the water column but still present in the bulk particles. This suggests particle-associated microbial communities that closely resemble the surrounding seawater. In contrast, the summer anticyclonic eddy demonstrated more pronounced differences between water column and particle-associated communities, as reflected by the constrained list of shared ASVs (Fig. 3B). Moreover, ASVs from particle-associated microbes, such as *Alteromonas*, showed high L2FC values in the anticyclone particles libraries. These ASVs, while enriched in the particles, were still present in some summer water samples. While mesoscale eddies can induce upwelling, decaying features such as the cyclone C2 [31], generally do not induce a biological response due to a diminished upwelling effect [11].

### Photoautotrophic community structure of seawater vs bulk particle libraries

We found that *Prasinococcales* were more dominant in the particle trap libraries of the AC2 compared to spring particle libraries and the EZ during both seasons (Fig. 4), and were indicators of the particle libraries (Fig. 5). Pigment analyses carried out at the same stations agree with these findings as they were mostly present at depths at or below the deep chlorophyll maximum [31]. Furthermore, *Bathycoccus* was differently abundant in the spring EZ (Fig. 6A), while *Ostreococcus* in the summer EZ (Fig. 6B), but were not identified as indicator of the particle libraries (Fig. 5). These results contrast with those of Cruz *et al.* [30], who identified *Bathycoccus* as an indicator of phytodetrital and fecal pellet aggregates in the Sargasso Sea, likely reflecting differences in sampling conditions. Amacher *et al.* [68] in the first DNA-based analysis of planktonic eukaryotes in particle trap libraries, found the prasinophytes *Micromonas*, *Ostreococcus,* and *Bathycoccus* in the particle communities, but not diatoms, despite their abundance in the water column. Treusch *et al.* [50] observed higher contribution of prasinophytes such as *Ostreococcus* during deep spring mixing events, hypothesized to be driven by dilution of grazing pressure [69]. This aligns with the deep mixing dynamics observed during the spring C2 at our study site, which may have similarly influenced community composition. In contrast, the unusual upwelling observed in the summer anticyclone likely promoted the growth and subsequent sedimentation of large diatoms, as evidenced by the significant contributions of the *R. setigera* ASV to the particle trap communities.

The relatively novel prasinophytes, *Chloropicon* and *Chloroparvula*, also contributed significantly to the particle communities, as identified by the indicator species analysis (Fig. 5). While their relative abundances were not dominant, their consistent presence across both spring and summer particle trap samples suggests they represent core taxa within these communities, despite differences in environmental conditions between the two seasons. Moreover, Bolaños *et al.* [70] found in the western North Atlantic that picocyanobacteria, pico-prasinophytes, and nanophytoplankton were dominant contributors to planktonic assemblages when large phytoplankton, such as diatoms, were generally infrequent. Blanco-Bercial *et al.* [71], in a three-year study of protist communities in the epi- and mesopelagic zones at BATS, found that *Pelagomonas*, *Ostreococcus*, *Bathycoccus,* and *Phaeocystis* were important taxa of the epipelagic autotrophic communities but also did not report on diatom ASVs.

Among the cyanobacteria, *Prochlorococcus* exhibited preference for the summer upper EZ and the deep-chlorophyll maximum, reflecting their adaptation to different light regimes [72]. Field observation of the same cruises, using clade-specific qPCR primers and flow cytometry, corroborated our findings, showing that *Prochlorococcus* clades were more abundant in the summer [32]. While *Prochlorococcus* ASVs also contributed to the summer anticyclone trap libraries, the prymnesiophyte *Phaeocystis* and the large centric diatom *Rhizosolenia* had greater contributions (Fig. 4). In contrast, *Synechococcus* was more abundant in the spring EZ (Supplementary Fig. 9), however, its summer contributions were reduced in both the water column (7%) and trap libraries (4%). These trends do not confirm the overrepresentation of *Synechococcus* and underrepresentation of *Prochlorococcus* in trap libraries previously reported by Amacher *et al.* [29]. Instead, our results show that *Synechococcus* exhibited a seasonal preference for spring conditions and showed some carryover to spring particle traps. These results may also explain the sedimentation of *Teleaulax* during both seasons (Fig. 5) but particularly into the spring particle traps (Fig. 6A). The mixotroph *Teleaulax* has been shown to grow fast under high-light conditions [73], and both *in silico* simulations [74] and culture-based experiments [75] suggest its abundance increases with greater presence of heterotrophic bacteria or *Synechococcus* due to predator–prey interactions. Although *Teleaulax* are important plastid donors to red-tide-forming dinoflagellates through indirect trophic interactions in coastal ecosystems [75, 76], their ecological role within pelagic ecosystems remains an open question.

### Sedimentation of Rhizosolenia after a mesoscale induced upwelling event

The *Rhizosolenia* genus consists of large-celled centric diatoms that exist in open ocean regions [77] typically dominated by small-celled phytoplankton [70]. Their opportunistic nature has been observed across diverse marine settings, including during stratified summer conditions in sediment traps samples [78] and water columns samples [79], and following sporadic nutrient mixing events [80]. *Rhizosolenia* export has also been linked to a cyclonic eddy-induced bloom off Hawai’i [81]. This chain-forming diatom genus has also been observed in net samples from the Sargasso Sea [77, 82], and found to form mats [83]. *Rhizosolenia* are an example of the large-celled diatoms hypothesized to play an outsized role in episodic primary production in this region [84]. Goldman and McGillicuddy [85] proposed that the annual new production in the Sargasso Sea could be driven by eddy-induced upwelling, which promotes the growth of large diatoms residing at the base of the EZ [84]. The significance of *Rhizosolenia* in the particle communities of the anticyclonic eddy experiencing wind-driven upwelling in its center, combined with the high particle flux, provides the first evidence of a sedimentation event of a large diatom in the Sargasso Sea as postulated by Goldman and McGillicuddy. These events highlight the capacity of *Rhizosolenia* to rapidly exploit transient nutrient pulses and disproportionately contribute to particle flux, exemplifying the broader ecological importance of rare, high-impact processes in these oligotrophic marine ecosystems [81, 86]. Increased diatom biomass is generally observed in response to mode-water eddies in the Sargasso Sea [11, 87], making the sedimentation of large-celled diatoms in our anticyclone a particularly notable observation.

We conclude that among the steady contribution of small-celled phytoplankton into the traps, episodic upwelling events can lead to a rapid increase of larger and opportunistic phytoplankton that make an important contribution not only to new but also to export production. This study provides evidence for the suggested effects of sporadic processes to mesoscale features [51], and shows how their combination can influence emergent patterns of carbon export. Although no unifying theory of the complexity of pelagic ecosystems and the role of sporadic events yet exists [81, 88], understanding these processes in seasonally stratified oceans such as the Sargasso Sea [8] is of special importance as oligotrophic regions make up most of open ocean and are thought to expand on a future warmer planet [1].

## Supplementary Material

ISMECOMMUN-D-24-00198_Suppl-Info_30-MAY-2025_ycaf083

## Data Availability

Data from the Trophic BATS project are available at https://www.bco-dmo.org/search/dataset/Trophic%20BATS (OCE-1030345, and OCE-1030476). 16S rRNA gene sequence data of the trap material as well as euphotic zone presented in this study are deposited in NCBI’s Sequence Read Archive (SRA) database under BioProject ID: PRJNA1111160.
