# Supplementary material for: In a sea of microbes, eddy events trigger diatom export in the Sargasso Sea
Source: ISME Commun. 2025 May 19;5(1):ycaf083. doi: 10.1093/ismeco/ycaf083 (PMC12202144; doi:10.1093/ismeco/ycaf083)
Supplement: ISMECOMMUN-D-24-00198_Suppl-Info_30-MAY-2025_ycaf083 [file ismecommun-d-24-00198_suppl-info_30-may-2025_ycaf083.pdf]

Supplementary Information for:
In a Sea of Microbes, Eddy Events Trigger Diatom Export in the Sargasso Sea

Marc Alec Fontánez Ortiz,[1,2,3] Francesca De Martini,[4] Susanne Neuer[1,3,5]*

[1]School of Life Sciences, Arizona State University, Tempe, AZ, 85287, USA
[2]School of Earth and Space Exploration, Arizona State University, Tempe, AZ, 85287, USA
[3]Center for Fundamental and Applied Microbiomics, The Biodesign Institute, Arizona State University, Tempe, AZ, 85287, USA
[4]Life Science Department, Mesa Community College, Mesa, AZ, 85202, USA
[5]School of Ocean Futures, Arizona State University, Tempe, AZ, 85287, USA

*Corresponding author. School of Ocean Futures, Walton Center for Planetary Health, Arizona State University, 777 E. University Dr., Tempe, AZ 85281, United States. E-mail: Susanne.Neuer@asu.edu

Content:

I. Detailed Materials and Methods
II. Supplementary Figures and Tables
III. Supplementary Text: *Clustering Fidelity: A Methodological Perspective*
IV. Supplementary References

I. Detailed Materials and Methods

Particle Interceptor Trap Processing:

The PITs array was fitted with acid-washed (10% HCl) 0.8 µm polycarbonate membrane filters at the bottom of each tube and filled with poisoned seawater brine (0.74% formalin final concentration; ca. 50g NaCl L^{-1} above ambient salinity), according to BATS standard protocols (Knap et al., 1997; http://bats.bios.edu/). Upon recovery, the caps and baffles from each tube were removed to siphon off the seawater that entered above the density layer of the poisoned brine. The remaining dense layer was left to filter through the membrane by opening the valve at the bottom. During this process, the caps for the tubes were placed back loosely to avoid ambient particles from entering the samples. After filtration, membranes were carefully removed from the tubes and placed onto acid-washed (10% HCl) glass Petri dishes to remove any swimmers (Buesseler et al., 2007) using sterile (70% EtOH) forceps and a dissection microscope on the ship. The particulate material attached to the membrane of each trap cone was rinsed with sterile-filtered seawater (0.2 µm/0.2 µm AcroPak 1000; Pall Corp) onto a 25 mm GF/F filter using a vacuum filtration system with cone-shaped funnels. The GF/F filters were placed in 1.5 mL cryovial tubes and immediately flash-frozen in liquid N_2.

DNA processing and Bioinformatics

After importing the FASTQ files, a median Q-score above 30 was chosen to avoid error rates larger than 0.1%. Primers were then trimmed using the q2-plugin *cutadapt*. Amplicon Sequence Variants (ASVs) from trimmed reads were prepared with q2-plugin *DADA2* (Callahan et al., 2016). Different sequence base positions were compared as truncation parameters to ensure sufficient read recovery during the denoising step. The best non-chimeric recovery was obtained at ca. 70% for most samples using position 243 (forward) and 166 (reverse) with a 12-base minimum overlap. ASVs were classified using the SILVA (v 138.1) database (Quast et al., 2013) and the PR^2 (v 15.0) database (Guillou et al., 2013) for the photoautotrophs. The PR^2 database includes plastidial 16S rRNA gene reference sequences from PhytoREF (Decelle et al., 2015) as of April 2021. The SILVA database was pre-processed using the q2-plugin *RESCRIPt* (Robeson et al., 2021) for standardized processing; this could not be done for PR^2. Contaminants, identified from environmental blanks prepared during aliquoting process after extraction, were removed. Furthermore, mitochondria, chloroplasts, and unassigned sequences from the SILVA-classified ASV table were removed, while the PR^2 -classified ASVs were filtered to only contain 16S rRNA gene sequences from plastids and cyanobacteria. The filtered libraries presented uneven sampling depth (see **Supplementary Text**), where seawater samples had more sequences than trap material. We used the q2-plugin *core-metrics-phylogenetic* for rarefaction and adjusted the library size using the smallest sample size with $\geq$ 1,000 sequences, resulting in the loss of three samples: a trap sample collected from BATS in spring 2012, a seawater sample collected from 20 m depth in AC2 in summer 2012, and a trap sample collected from BATS in summer 2012 **(Supplementary Table 1)**. Saturation curves were used to validate the effects of rarefaction depth on diversity metrics **(Supplementary Figures 2–3)**. After applying rarefaction, we visualized alpha-diversity proxies (**Supplementary Figures 4–5**) determining richness (Observed Features), phylogenetic richness (Faith PD), evenness (Pielou's Evenness) and within-sample diversity (Shannon's Entropy).

Statistical Analysis: Statistical modeling was performed within R statistical software (v 4.3.2) by creating an R object of the rarefied libraries using the *phyloseq* package (v 1.46.0) (McMURDIE & Holmes, 2011). For statistical analysis that required randomization, eight random values were repeatedly used as seed for reproducibility. The rarefied ASV tables in the *phyloseq* objects were used to prepare a non-metric multidimensional scaling (NMDS) ordination based on Bray-Curtis dissimilarity distances. The differences associated with sample types (seawater and trap material), season (spring and summer) as well as depth (20 m, DMC, and 150 m) were visualized.

Overdispersion Analysis:

To evaluate overdispersion in our dataset, we assessed the relationship between mean abundance and variance using the *phyloseq* (v 1.46.0) and *DESeq2* (v 1.42.0) packages in R (v 4.3.2). After estimating size factors and dispersions, we calculated the mean normalized counts and variances for each taxon, log-transformed the values, and plotted them. A dashed line representing the Poisson expectation (Mean Variance=Mean) was added using the *abline* function in R, specifying a slope of 1 and intercept of 0 to represent the linear relationship expected under Poisson assumptions. A locally estimated scatterplot smoothing (LOESS) line was generated using the *loess.smooth* function, which fits a non-parametric LOESS curve to visualize deviations from Poisson distribution expectation.

Alpha–Rarefaction:

Alpha diversity metrics were subject to an initial alpha-rarefaction analysis to evaluate diversity across even sampling depths. Median values from the alpha-rarefaction metrics were calculated using rarefied ASV tables generated through the q2-plugin *alpha-rarefaction* in QIIME2, applying 10 iterations for rarefaction at multiple depths. The maximum sampling depth was set to a value equal to the sequencing depth that retained most samples, but that was higher than 1,000. Observed Features (as a measure of richness) and Shannon's Index (as a measure of diversity) were used as the metrics for alpha-rarefaction (**Supplementary Figures 2–3**).

Alpha–Diversity Analysis:

Visual inspection of data normality was done via histograms, and statistical inspection was conducted using the Shapiro-Wilk test. The homogeneity of variances of the residuals was verified using Levene's test. Since not all data followed a normal distribution, non-parametric assumptions were applied, and the Kruskal-Wallis H test was used to determine whether significant differences in medians existed between predetermined groups. Groups were defined by depth (20 m, DCM, and PITs) and season (spring and summer). Post hoc pairwise comparisons were conducted using the Dunn's Multiple Comparisons test. To control for the False Discovery Rate (FDR) and reduce Type I errors, p-values were adjusted using the Benjamini-Hochberg method. Plots were created to visualize alpha diversity metrics were plotted as plotted as violin plots overlaid to box and whiskers plots to assess group significance, distribution, and variation among sample groups. The *ggbetweenstats* function from the *ggstatsplot* package (v 0.9.1) in R was used for statistical analysis and simultaneous plotting. Additional custom visualizations were created using the *ggplot2* (v 3.3.5) and *reshape* (v 0.8.8) packages in R (v 4.0.5).

To assess the influence of different normalization techniques on Bray-Curtis dissimilarities, we compared pairwise dissimilarities between the rarefied table and transformed table using the *phyloseq* object and the *vegan* (v 2.6-4) package. Pearson correlation coefficients (R^2) and Root Mean Square Error (RMSE) quantified the alignment of calculated Bray-Curtis dissimilarity, while scatterplots generated with *ggplot2* (v 3.3.5) visualized the relationships between "Actual BC" from the rarefied table and "Calculated BC."

Assessing clustering robustness across normalization techniques:

We compared clustering robustness using Approximately Unbiased (AU) p-values and Adjusted Rand Index (ARI) metrics calculated with the *pvclust* (v 2.2-0) and *mclust* (v 6.0.0) packages in R. AU p-values, visualized through violin plots generated with *ggstatsplot* (v 0.9.1), highlighted variability in clustering confidence across normalization techniques. ARI values, computed relative to the rarefied table, quantified alignment in cluster memberships using a tree height cutoff of 0.9 based on the Bray-Curtis dissimilarity index.

Indicator Species Analysis

The indicator value was calculated as follows:

$$IndVal_{ij} = \sqrt{100(A_{ij} * B_{ij})}$$

Where A_{ij} is the specificity, i.e., the abundance of species i in group j, and B_{ij} is the fidelity, i.e., frequency by which species i appears in group j. Indictor values are highest when taxa are exclusively found in one of the *a priori* groups while expressing high relative abundance within that same group (Severns & Sykes, 2020). A Monte Carlo permutational test (nperm = 9999) was also added using the *how* function from the *indicspecies* dependency, *permute* (v 1.9-5). This function helped assess the statistical significance of each indicator value. To report the group-wise indicator values, the *p-value* was adjusted for multiple testing issues, as advised by De Cáceres et al. (2010), using the Benjamini-Hochberg procedure, and only taxa with an adjusted $p < 0.05$ were chosen.

II. Supplementary Figures and Tables

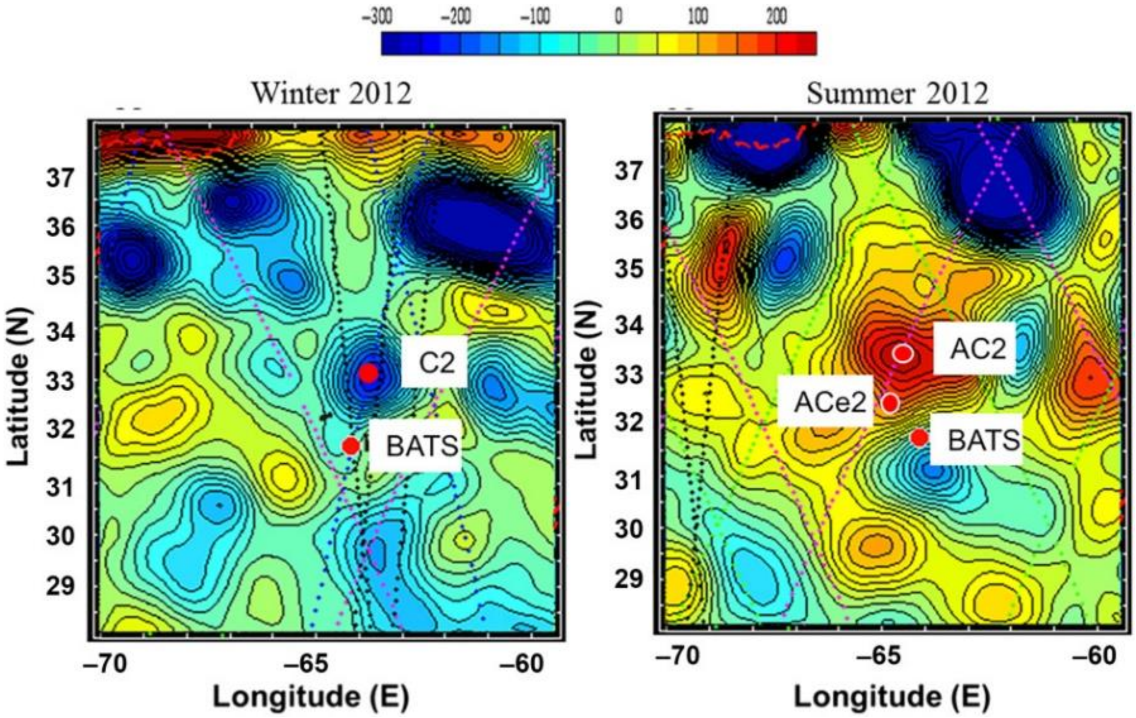

Supplementary Figure 1: This study is part of the Trophic-BATS project on the effect of mesoscale eddies to trophic interactions and carbon export in the oligotrophic Sargasso Sea (Cotti-Rausch et al., 2016; De Martini et al., 2018). This study explores plankton community export, focusing on the prokaryotic and photoautotrophic assemblages collected from the euphotic zone and Particle Interceptor Traps at 150 m. The Sargasso Sea is characterized by strong meridional gradients (Siegel, 1990; Sweeney et al., 2003), having seasonal mixing events in the northernmost part and permanent stratification in the south (Cianca et al., 2012; Nelson et al., 2004). This study's location lies near the center of this gradient, where mesoscale eddies have been suggested as important and dominant features that induce biological responses by producing upwelling events in the upper ocean (Cianca et al., 2012; McGillicuddy et al., 2001). Adapted from "Clade and Strain Specific Contributions of Synechococcus and Prochlorococcus to Carbon Export in the Sargasso Sea" by De Martini F., Neuer S., Hamil D. et al. *Limnol Oceanogr*, 2018;**63**. https://doi.org/10.1002/lno.10765. Copyright 2017 Association for the Sciences of Limnology and Oceanography. Reprinted with permission from John Wiley and Sons.

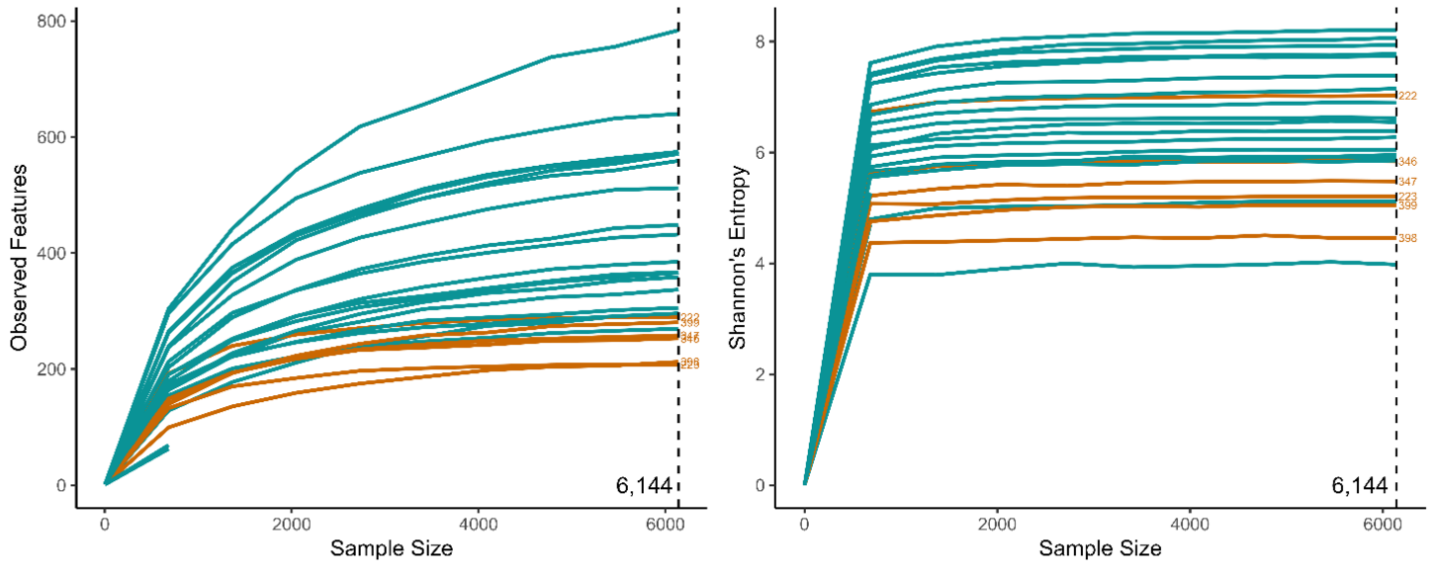

Supplementary Figure 2: Saturation curves of prokaryotes, showing median alpha-diversity estimators for Observed Features (left) and Shannon's Entropy (right) across multiple iteration as function of sequencing depth for 16S rRNA gene amplicon libraries. Curves are color-coded to differentiate between seawater (blue) and bulk trap material (brown). The black dashed vertical line represents the library size depth (6,144) used to rarefy the SILVA (v.138.1)-classified ASV table.

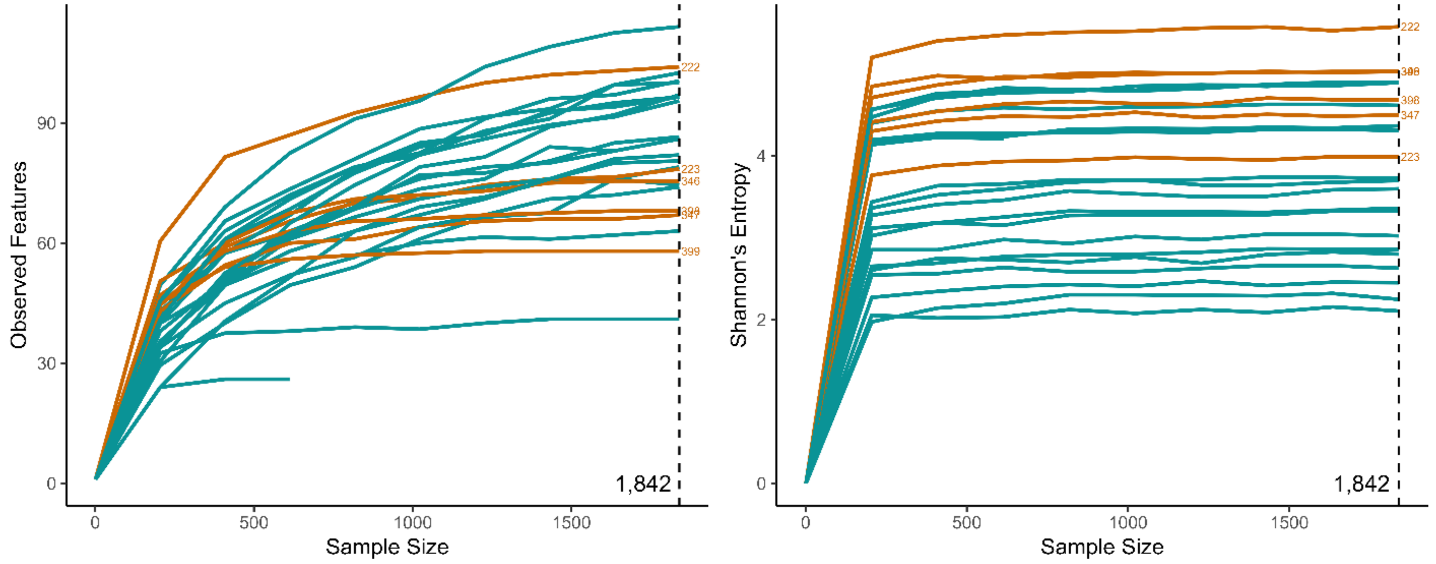

Supplementary Figure 3: Saturation curves of photoautotrophs (plastids and cyanobacteria), showing median alpha-diversity estimators for Observed Features (left) and Shannon's Entropy (right) across multiple iteration as function of sequencing depth for 16S rRNA gene amplicon libraries. Curves are color-coded to differentiate between seawater (blue) and bulk trap material (brown). The black dashed vertical line represents the library size depth (1,842) used to rarefy the PR2 (v.5.0.0)-classified ASV table.

				Prokaryotes			
Station	Sample ID	Depth	Sequence Counts	Observed Features	Faith's PD	Pielou's Evenness	Shannon's Entropy
C2	173	20	37995	338	41.96925	0.602257	5.059487
	175	80	51116	454	55.25978	0.744998	6.575757
	202	20	49064	295	41.42742	0.492122	4.037652
	205	100	55807	574	53.22728	0.84065	7.704477
	222	150	11099	290	60.0255	0.859245	7.028549
	223	150	8250	208	48.3058	0.676548	5.209716
BATS (B3)	232	20	35858	370	42.77526	0.739975	6.313006
	234	80	56788	514	47.12434	0.789613	7.110955
	253	20	31018	304	39.34914	0.717432	5.917328
	258	100	31806	643	67.77254	0.876815	8.179521
	283†	150	1549	–	–	–	–
	284*	150	–	–	–	–	–
AC2	288†	20	10	–	–	–	–
	290*	85	–	–	–	–	–
	316	20	31600	268	35.19064	0.747384	6.028467
	319	110	69393	810	77.40179	0.840263	8.118436
	346	150	11218	252	58.57711	0.734832	5.861963
	347	150	11511	258	58.949	0.686161	5.496991
Ace2	348	20	54347	362	46.23141	0.701743	5.964708
	351	100	38611	430	54.76983	0.789326	6.905176
	376	20	58222	376	46.0813	0.683514	5.847182
	379	100	43031	560	59.87039	0.871793	7.958844
	398	150	17515	210	44.83831	0.58086	4.480897
	399	150	14899	281	57.03769	0.620421	5.046772
BATS (B4)	406	20	56840	358	40.86014	0.756662	6.419383
	409	95	39092	563	62.83856	0.845531	7.725613
	438	20	37490	301	39.748	0.802927	6.610999
	441	100	53130	585	63.92832	0.798901	7.343735
	442*	150	–	–	–	–	–
	443†	150	769	–	–	–	–

Supplementary Table 1: Sample information, feature counts, and alpha-diversity indices of prokaryotic 16S rRNA genes from samples collected in a spring cyclonic eddy (C2), a summer anticyclonic eddy center (AC2) and edge (Ace2) as well as from the Bermuda Atlantic Time-series Study station (BATS) in spring (B3) and summer (B4). Samples from 150 m are from the Particle Interceptor Traps (PITs). † Low sample size, *Missing sample, –No value

Station	Sample ID	Sample Type	Depth	Sequence Counts	Photoautotrophs Observed Features	Faith's PD	Pielou's Evenness	Shannon's Entropy
C2	173	Seawater	20	37995	81	7.538885	0.409081	2.59351
	175	Seawater	80	51116	98	8.69954	0.56174	3.715745
	202	Seawater	20	49064	67	6.239197	0.345457	2.095573
	205	Seawater	100	55807	87	7.896023	0.68605	4.420182
	222	PITs	150	11099	99	9.439521	0.841656	5.579636
	223	PITs	150	8250	75	7.609309	0.638177	3.975091
BATS (B3)	232	Seawater	20	35858	101	10.38172	0.555761	3.700374
	234	Seawater	80	56788	83	9.359482	0.546045	3.481055
	253	Seawater	20	31018	88	9.187102	0.504163	3.256609
	258	Seawater	100	31806	40	7.220285	0.807255	4.296152
	283†	PITs	150	1549	–	–	–	–
	284*	PITs	150	–	–	–	–	–
AC2	288†	Seawater	20	10	–	–	–	–
	290*	Seawater	85	–	–	–	–	–
	316	Seawater	20	31600	85	9.970722	0.435593	2.791888
	319	Seawater	110	69393	114	9.259575	0.724378	4.949597
	346	PITs	150	11218	76	11.47555	0.80912	5.055323
	347	PITs	150	11511	67	9.12844	0.74075	4.493453
Ace2	348	Seawater	20	54347	83	7.887139	0.356934	2.275466
	351	Seawater	100	38611	75	9.279173	0.4793	2.985471
	376	Seawater	20	58222	103	10.91511	0.372428	2.490243
	379	Seawater	100	43031	63	8.846757	0.771476	4.61133
	398	PITs	150	17515	69	9.248262	0.770927	4.709228
	399	PITs	150	14899	58	7.487575	0.858911	5.031487
BATS (B4)	406	Seawater	20	56840	95	10.13328	0.430774	2.830126
	409	Seawater	95	39092	102	9.819854	0.725778	4.842702
	438	Seawater	20	37490	95	11.42932	0.548732	3.60509
	441	Seawater	100	53130	102	9.20507	0.731994	4.884177
	442*	PITs	150	–	–	–	–	–
	443†	PITs	150	769	–	–	–	–

Supplementary Table 2: Sample information, feature counts, and alpha-diversity indices of photoautotrophic (plastids and cyanobacteria) 16S rRNA genes from samples collected in a spring cyclonic eddy (C2), a summer anticyclonic eddy center (AC2) and edge (Ace2) as well as from the Bermuda Atlantic Time-series Study station (BATS). Samples from 150 m are from the Particle Interceptor Traps (PITs). †Low sample size, *Missing sample, –No value.

	Seawater			PITs			Seawater & PITs		
Predictor	R^2	p-value	FDR	R^2	p-value	FDR	R^2	p-value	FDR
Season	0.229	0.003**	0.009**	0.431	0.067	0.1	0.447	0.001***	0.001***
Depth	0.259	0.004**	0.009**	–	–	–	0.359	0.001***	0.001***
Location	0.371	0.030*	0.042*	0.816	0.067	0.1	0.617	0.001***	0.001***
Sample Type	–	–	–	–	–	–	0.220	0.001***	0.001***
Depth (Spring)	0.385	0.002*	0.009*	–	–	–	0.523	0.001***	0.001***
Depth (Summer)	0.483	0.012*	0.0245*	–	–	–	0.584	0.001***	0.001***
Location (Spring)	0.199	0.190	0.222	–	–	–	0.422	0.007***	0.007***
Location (Summer)	0.178	0.541	0.541	0.896	0.333	0.333	0.594	0.002***	0.001***
Sample Type (Spring)	–	–	–	–	–	–	0.312	0.029*	0.029*
Sample Type (Summer)	–	–	–	–	–	–	0.356	0.004**	0.008**

Supplementary Table 3: Pairwise permutational multivariate analysis of variance (PERMANOVA) based on Bray-Curtis dissimilarities from rarefied abundances of prokaryotic 16S rRNA genes. This table presents the effects of seasonality (spring & summer), depth (20m, DCM, & PITs), location (C2, B3, AC2, Ace2, and B4), and sample type (Seawater & PITs) on prokaryotic community composition. The analysis was conducted separately for seawater, PITs, and combined datasets. Comparisons were restricted to sample groups with $n > 4$. Significance levels are indicated with asterisks (FDR-corrected p-values: *$p < 0.05$, **$p < 0.01$, ***$p < 0.001$).

For seawater samples, season explained 22.9% of the variation ($R^2 = 0.229$; FDR = 0.009), location explained 37.1% ($R^2 = 0.371$; FDR = 0.042), and depth explained 25.9% ($R^2 = 0.259$; FDR = 0.009), all of which were significant predictors of community composition. Intra-seasonal variations for seawater were primarily explained by depth during spring (38.5%, $R^2 = 0.385$; FDR = 0.009) and summer (48.3%, $R^2 = 0.483$; FDR = 0.0245), while location was not a significant driver of variability within seasons. For PITs samples, no significant predictors (season, depth, or location) explained differences in prokaryotic community composition, although location (81.6%, $R^2 = 0.816$; FDR = 0.1) showed marginal significance. In the combined seawater and PITs dataset, season explained 44.7% of the variation ($R^2 = 0.447$; FDR = 0.001), depth explained 35.9% ($R^2 = 0.359$; FDR = 0.001), and location explained 61.7% ($R^2 = 0.617$; FDR = 0.001), with sample type also emerging as a significant factor, explaining 22.0% of the variation ($R^2 = 0.220$; FDR = 0.001).

	Seawater			PITs			Seawater & PITs		
Predictors	R^2	p-value	FDR	R^2	p-value	FDR	R^2	p-value	FDR
Season	0.327	0.001***	0.004**	0.501	0.067	0.1	0.471	0.001***	0.001**
Depth	0.279	0.001***	0.003***	–	–	–	0.357	0.001***	0.001***
Location	0.421	0.023*	0.032*	0.713	0.067	0.1	0.568	0.001***	0.001***
Sample Type	–	–	–	–	–	–	0.675***	0.001***	0.001***
Depth (Spring)	0.376	0.004**	0.009*	–	–	–	0.491	0.001***	0.001***
Depth (Summer)	0.587	0.011*	0.019*	–	–	–	0.656	0.001***	0.001***
Location (Spring)	0.169	0.336	0.392	–	–	–	0.367	0.045*	0.051
Location (Summer)	0.129	0.661	0.661	0.619	0.333	0.333	0.446	0.092	0.092
Sample Type (Spring)	–	–	–	–	–	–	0.265	0.047*	0.047*
Sample Type (Summer)	–	–	–	–	–	–	0.308	0.007*	0.014*

Supplementary Table 4: Pairwise permutational multivariate analysis of variance (PERMANOVA) based on Bray-Curtis dissimilarities from the rarefied abundances of photoautotrophic (plastids and cyanobacteria) 16S rRNA genes. This table presents the effects of seasonality (spring & summer), depth (20m, DCM, & PITs), location (C2, B3, AC2, Ace2, and B4), and sample type (Seawater & PITs) on community composition. The analysis was conducted separately for seawater, PITs, and combined datasets. Comparisons were restricted to sample groups with n > 4. Significance levels are indicated with asterisks (FDR-corrected p-values: *p < 0.05, **p < 0.01, ***p < 0.001).

For seawater samples, season explained 32.7% of the variation ($R^2 = 0.327$; FDR = 0.004), depth explained 27.9% ($R^2 = 0.279$; FDR = 0.003), and location explained 42.1% ($R^2 = 0.421$; FDR = 0.032), all of which were significant predictors of community composition. Within-season variations for seawater were significantly driven by depth in spring (37.6%, $R^2 = 0.376$; FDR = 0.009) and summer (58.7%, $R^2 = 0.587$; FDR = 0.019). Location was not a significant predictor in spring (16.9%, $R^2 = 0.169$; FDR = 0.392) or summer (12.9%, $R^2 = 0.129$; FDR = 0.661). For PITs samples, no significant predictors (season, depth, or location) explained differences in plastid and cyanobacterial community composition, although location (71.3%, $R^2 = 0.713$; FDR = 0.1) showed marginal significance.

In the combined seawater and PITs dataset, season explained 47.1% of the variation ($R^2 = 0.471$; FDR = 0.001), depth explained 35.7% ($R^2 = 0.357$; FDR = 0.001), and location explained 56.8% ($R^2 = 0.568$; FDR = 0.001). Additionally, sample type emerged as a significant factor, explaining 67.5% of the variation ($R^2 = 0.675$; FDR = 0.001). Within-season analyses revealed that depth explained variation during spring (49.1%, $R^2 = 0.491$; FDR = 0.001) and summer (65.6%, $R^2 = 0.656$; FDR = 0.001), while location significantly explained variation in spring (36.7%, $R^2 = 0.367$; FDR = 0.051) but not summer (44.6%, $R^2 = 0.446$; FDR = 0.092). Sample type was a significant predictor in spring (26.5%, $R^2 = 0.265$; FDR = 0.047) and summer (30.8%, $R^2 = 0.308$; FDR = 0.014).

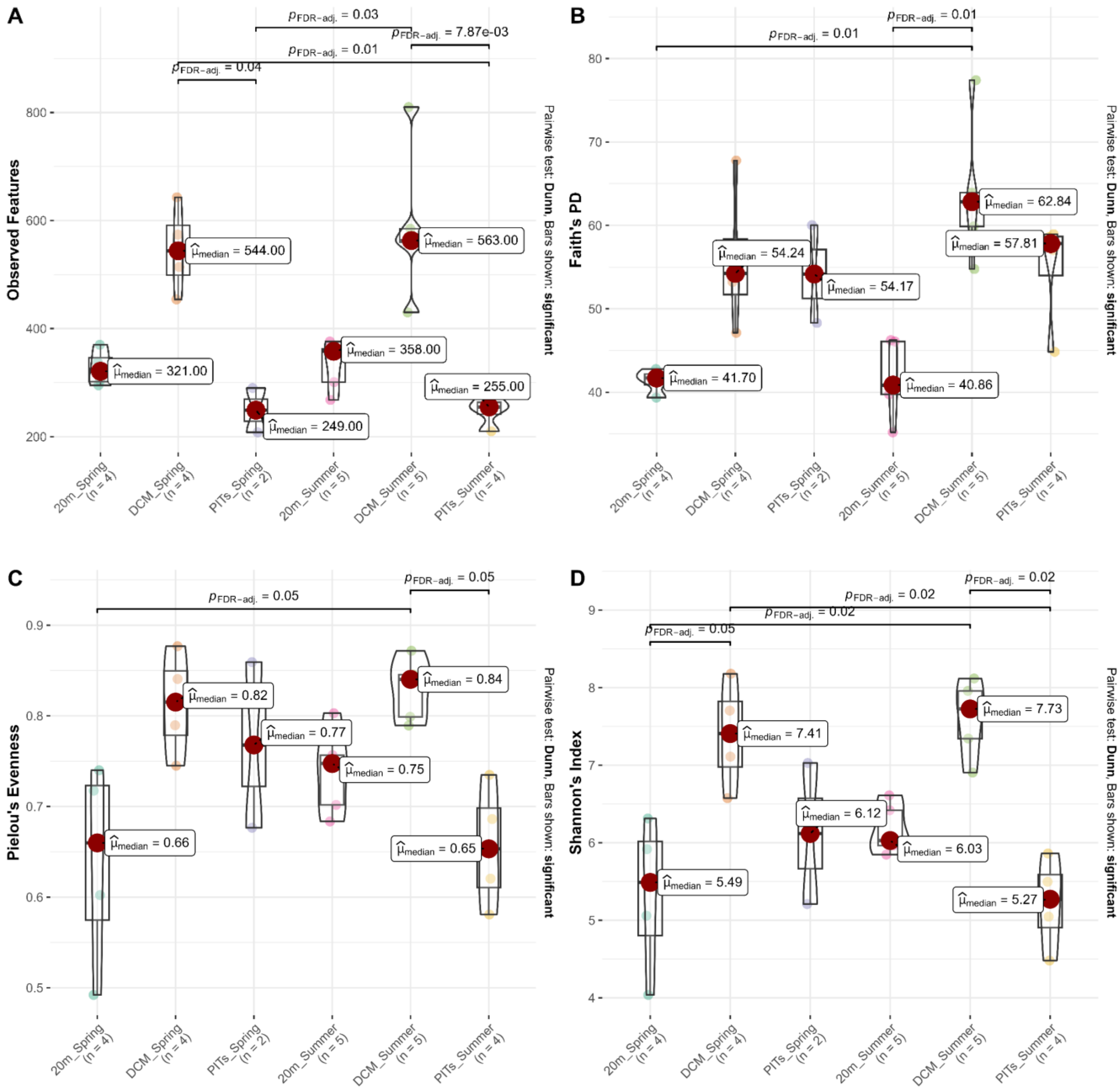

Supplementary Figure 4: Alpha-diversity metrics of the 16S rRNA gene amplicon libraries targeting prokaryotes plotted as violin plots overlaid to box and whiskers showing both distribution and variations of a) Observed Features, b) Faith's Phylogenetic Distance, c) Pielou's Evenness, and d) Shannon's Entropy as function of depth across Spring and Summer 2012, encompassing samples from the upper 20 m, the deep chlorophyl maximum (DCM), and bulk particle material from 150 m depth (PITs). Pairwise comparisons were performed using Dunn's post hoc test with FDR-corrected p-values, shown for statistically significant differences. Metrics were plotted as a function of depth for Spring 2012 and Summer 2012. Samples represent the upper 20m, Deep Chlorophyl Maxima (DCM) and trap material collected at 150m (PITs). The FDR-correct *p-values* represent pairwise group significance of samples that are significantly differences (*p < 0.05, **p < 0.01, ***p < 0.001). The median values of each group are labeled within the plots.

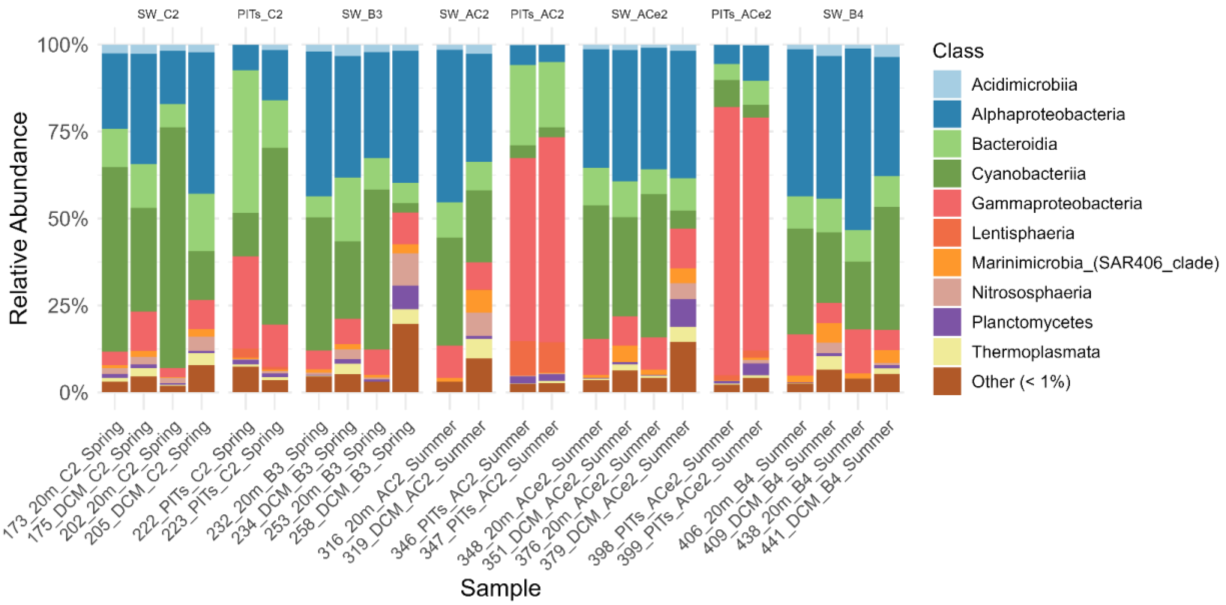

Supplementary Figure 5: Relative abundance of prokaryotic 16S rRNA gene amplicons collapsed at the Class-level of the rarefied table. Classes that represented less than 1% (i.e., taxa with < 20 read counts) of the table were collapsed to the "Other" category. Bars are grouped by eddy (C2, B3, AC2, Ace2 and B4) and sample type, seawater (SW) and particle trap material (PITs). Samples C2 and B3 were collected in Spring 2012 and samples AC2, Ace2 and B4 were collected in Summer 2012.

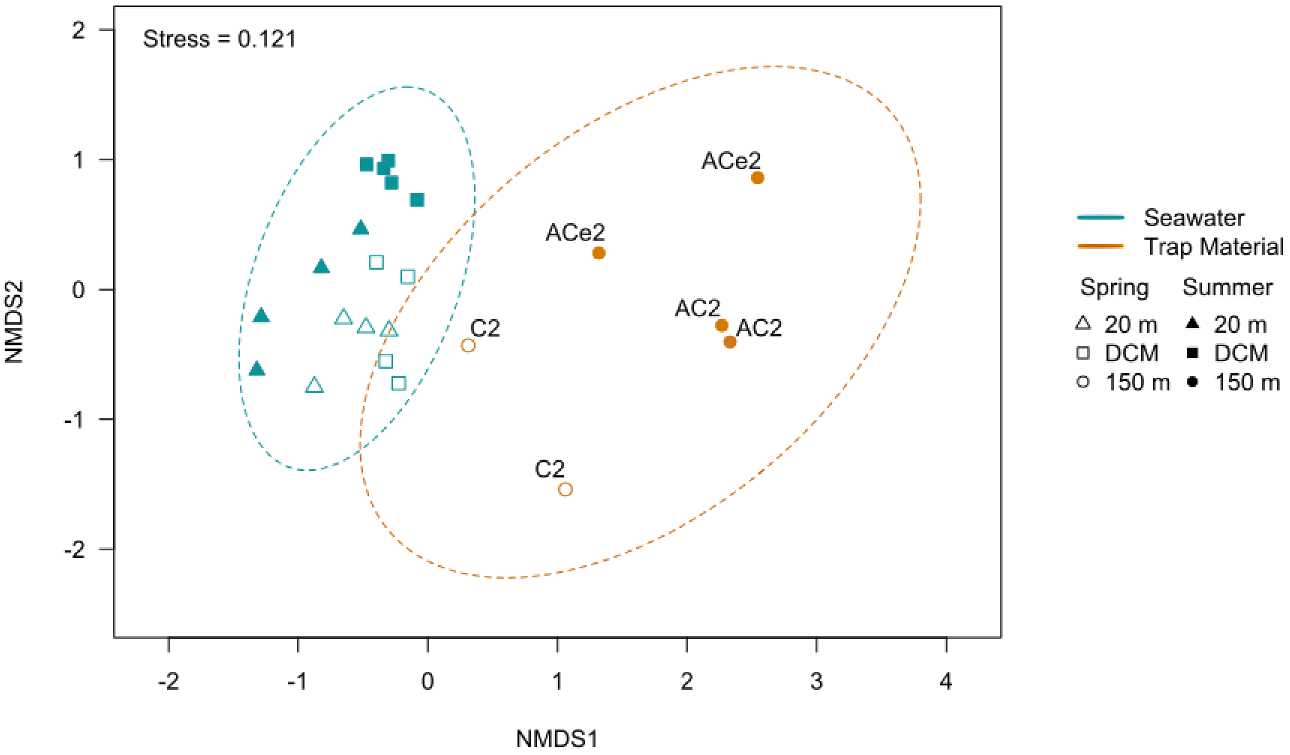

Supplementary Figure 6: Non-metric Multidimensional Scaling (NMDS) ordination of Bray-Curtis dissimilarity based on the rarefied table depicting the compositional differences of the prokaryotic community in seawater (blue) and trap material (brown) during spring (filled symbols) and summer (unfilled symbols) samples collected at four different depths (symbol shape). Dashed lines represent 95% confidence ellipses. Trap material labels correspond to the spring cyclonic eddy (C2) and the summer anticyclone center (AC2) and edge (Ace2).

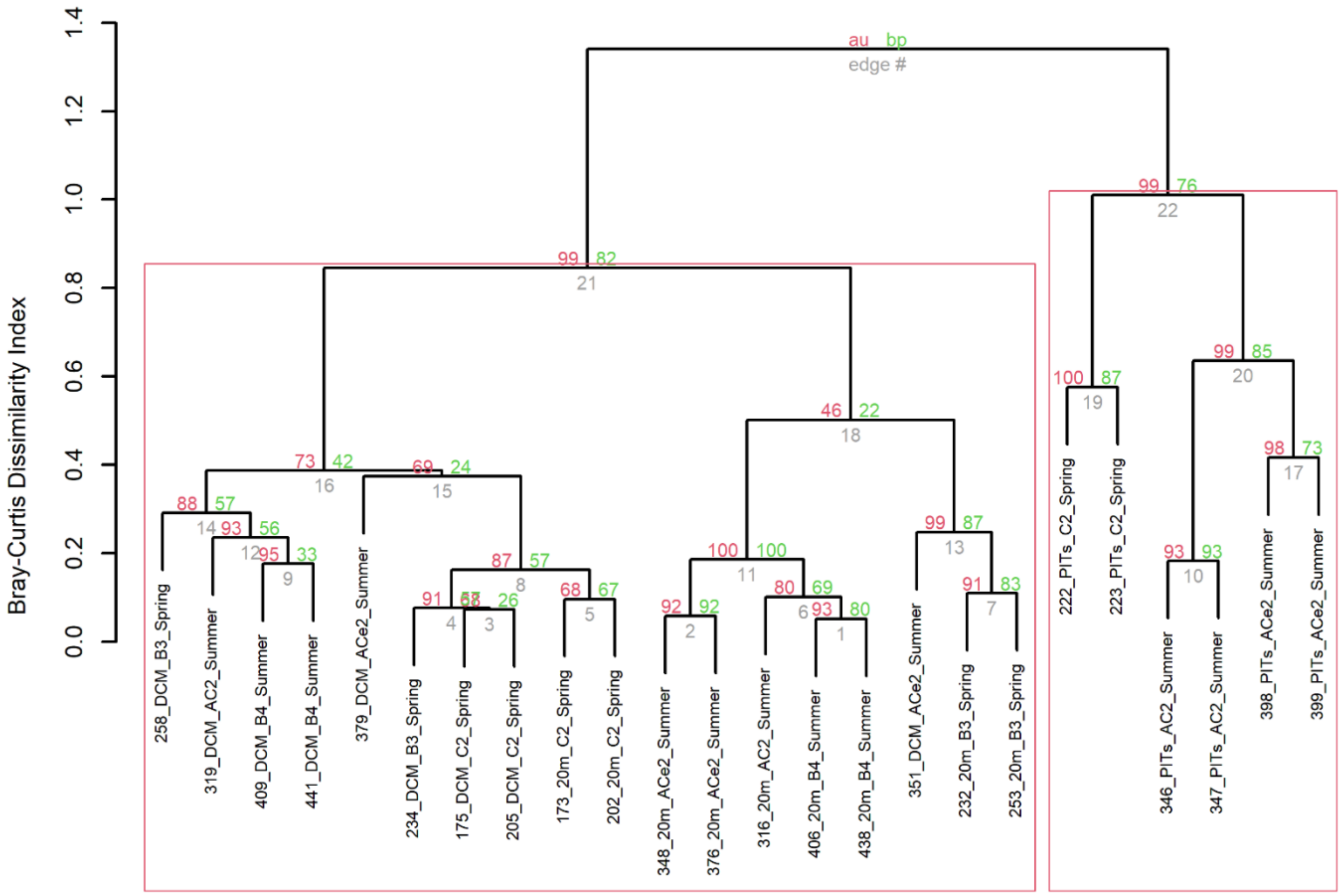

Supplementary Figure 7: The Unweighted Pair Group Method with Arithmetic Mean (UPGMA) dendrogram was constructed using Bray-Curtis dissimilarities based on a log(x+1)-transformed rarefied prokaryotic 16S rRNA gene amplicon data. This transformation reduces the influence of dominant species while preserving relative differences between samples (see **Supplementary Text**). Cluster significance was assessed using bootstrapping with 1,000 iterations at ≥ 95% confidence level, implemented via the *pvclust* package in R. Red rectangles highlight significant clusters with Approximately Unbiased (AU) p-values (AU ≥ 95%), indicating statistically significant similarity (red numbers). Bootstrap Probability (BP) values (green numbers) provide a measure of cluster stability across iterations. Edge numbers (grey values) correspond to branch divisions in the dendrogram, representing hierarchical clustering relationships. This dendrogram was used to depict sample differences in **Figure 1** in the main text.

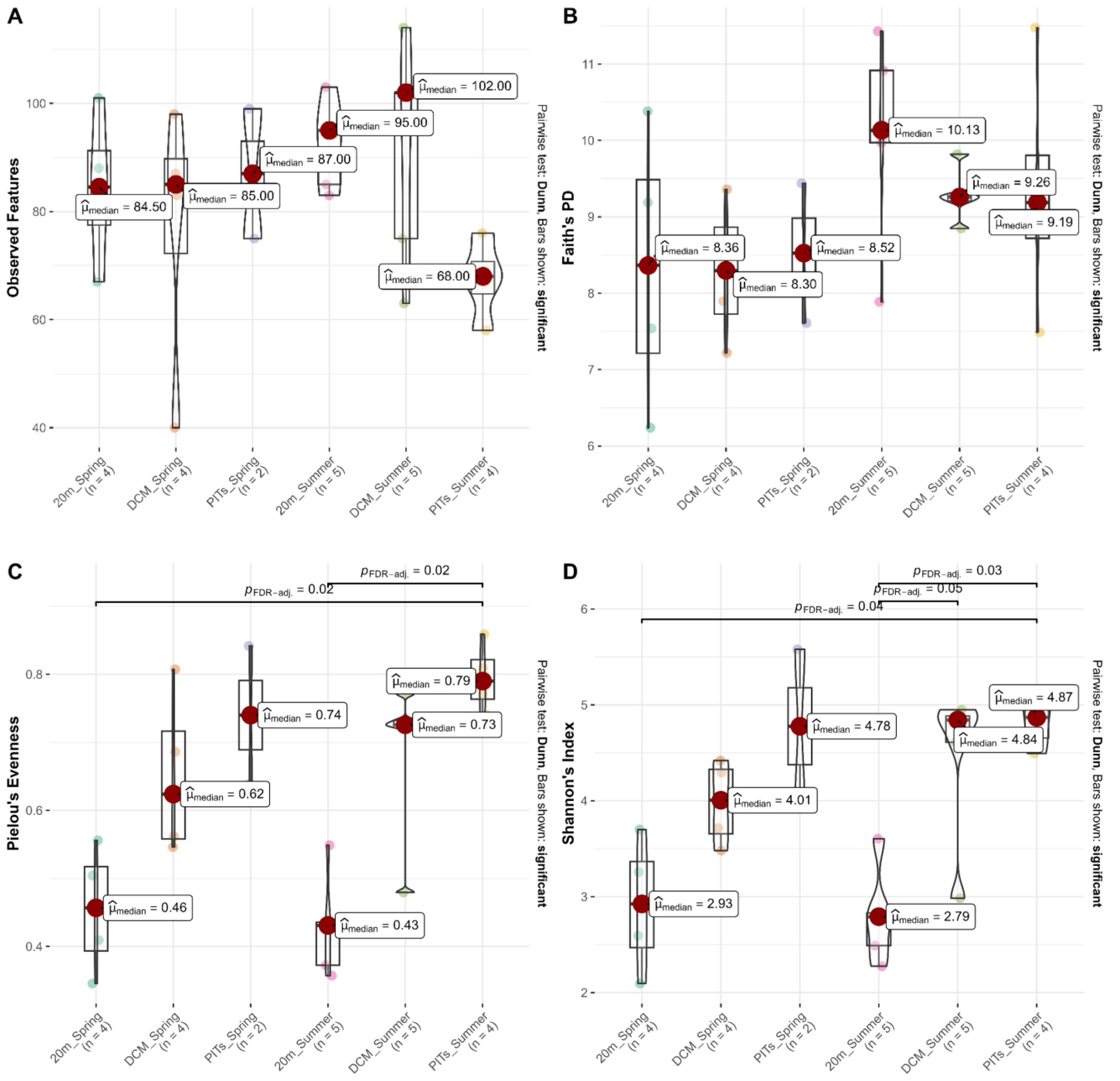

Supplementary Figure 8: Alpha-diversity metrics of the 16S rRNA gene amplicon libraries targeting photoautotrophs (plastids and cyanobacteria) plotted as violin plots overlaid to box and whiskers showing both distribution and variations of a) Observed Features, b) Faith's Phylogenetic Distance, c) Pielou's Evenness, and d) Shannon's Entropy as function of depth across Spring and Summer 2012, encompassing samples from the upper 20 m, the deep chlorophyll maximum (DCM), and bulk particle material from 150 m depth (PITs). Pairwise comparisons were performed using Dunn's post hoc test with FDR-corrected p-values, shown for statistically significant differences. Metrics were plotted as a function of depth for Spring 2012 and Summer 2012. Samples represent the upper 20m, Deep Chlorophyll Maxima (DCM) and trap material collected at 150m (PITs). The FDR-correct *p-values* represent pairwise group significance of samples that are significantly differences (*p < 0.05, **p < 0.01, ***p < 0.001). The median values of each group are labeled within the plots.

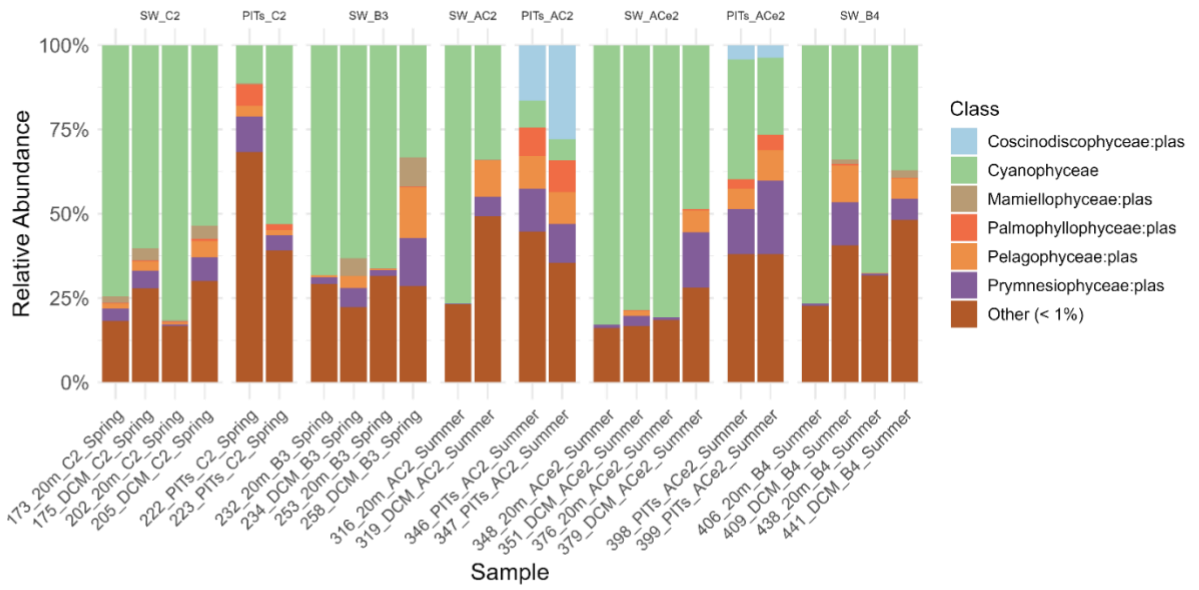

Supplementary Figure 9: Relative abundance of photoautotrophs (plastids and cyanobacteria) 16S rRNA gene amplicons collapsed at the Class-level of the rarefied table. Classes that represented less than 1% (i.e., taxa with < 20 read counts) of the table were collapsed to the "Other" category. Bars are grouped by eddy (C2, B3, AC2, Ace2 and B4) and sample type, seawater (SW) and particle trap material (PITs). Samples C2 and B3 were collected in Spring 2012 and samples AC2, Ace2 and B4 were collected in Summer 2012.

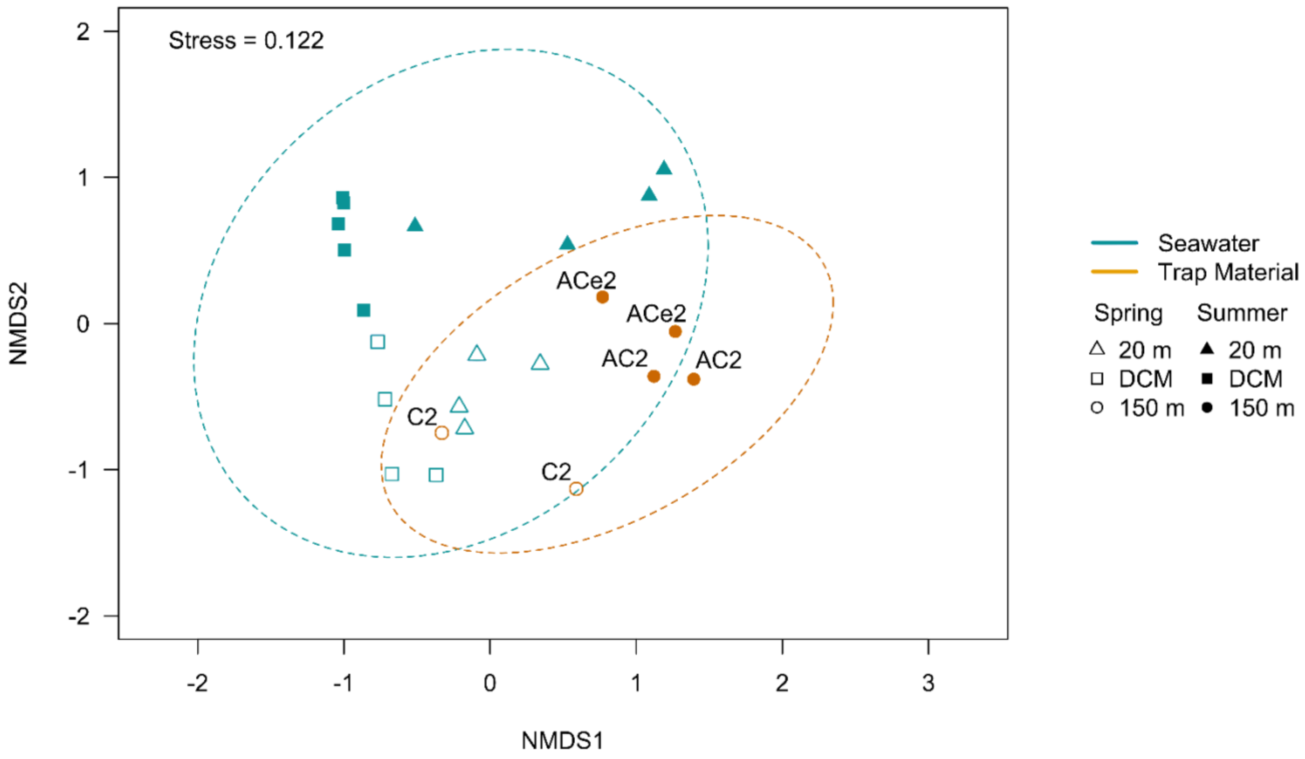

Supplementary Figure 10: Non-metric Multidimensional Scaling (NMDS) ordination of Bray-Curtis dissimilarity based on the rarefied table depicting the compositional differences of the photoautotrophic (plastid and cyanobacteria) community in seawater (blue) and trap material (brown) during spring (filled symbols) and summer (unfilled symbols) samples collected at four different depths (symbol shape). Dashed lines represent 95% confidence ellipses. Trap material labels correspond to the spring cyclonic eddy (C2) and the summer anticyclone center (AC2) and edge (Ace2).

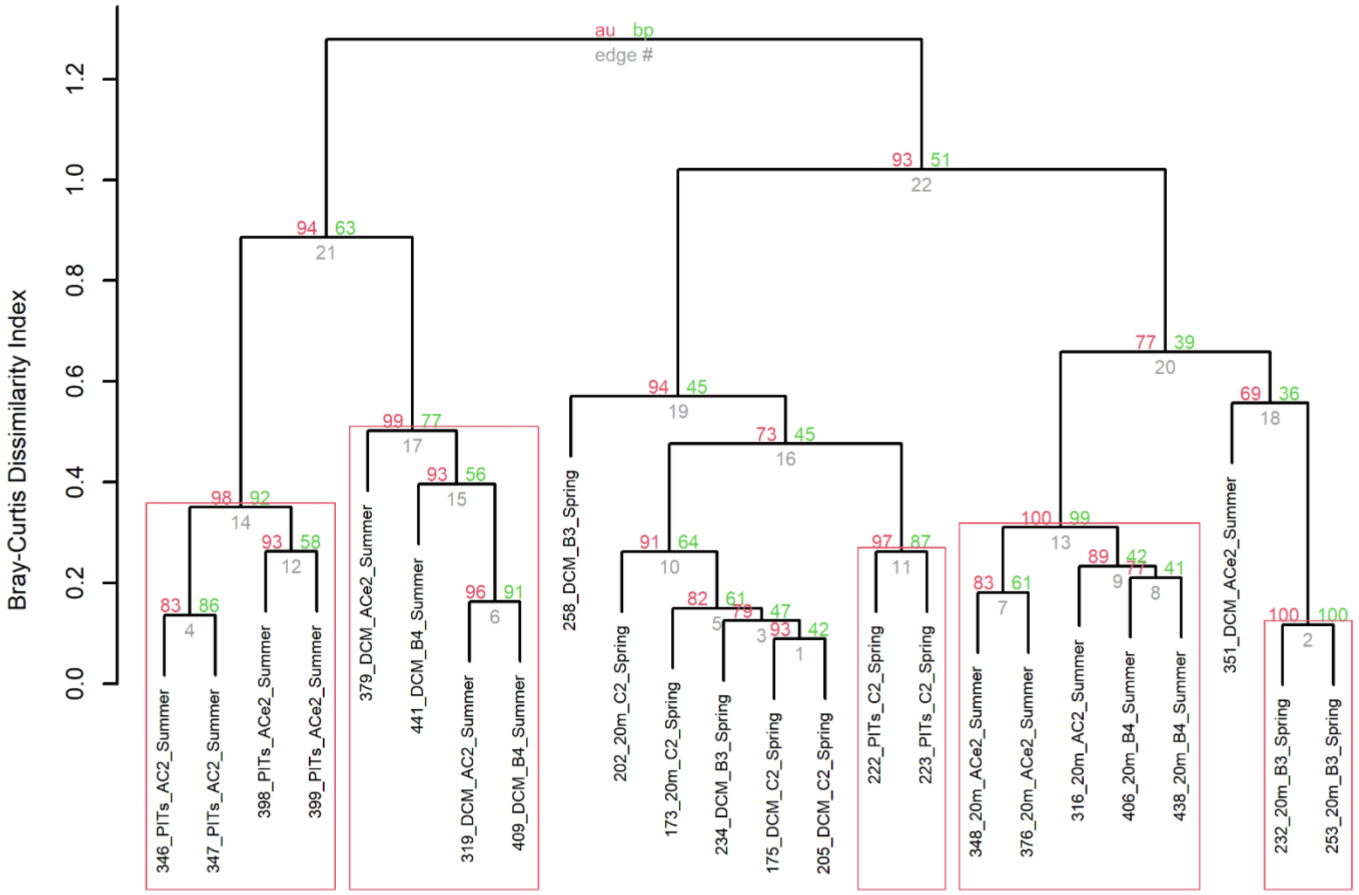

Supplementary Figure 11: The Unweighted Pair Group Method with Arithmetic Mean (UPGMA) dendrogram was constructed using Bray-Curtis dissimilarities based on a log(x+1)-transformed rarefied plastid and cyanobacterial 16S rRNA gene amplicon data. This transformation reduces the influence of dominant species while preserving relative differences between samples (see **Supplementary Text**). Cluster significance was assessed using bootstrapping with 1,000 iterations at ≥ 95% confidence level, implemented via the *pvclust* package in R. Red rectangles highlight significant clusters with Approximately Unbiased (AU) p-values (AU ≥ 95%), indicating statistically significant similarity (red numbers). Bootstrap Probability (BP) values (green numbers) provide a measure of cluster stability across iterations. Edge numbers (grey values) correspond to branch divisions in the dendrogram, representing hierarchical clustering relationships. This dendrogram was used to depict sample differences in **Figure 4** in the main text.

Generalist ASVs	PITs Spring	PITs Summer	SW Spring	SW Summer	IndVal	p–value	FDR p–value
Prochlorococcus_MIT9313	1	1	1	1	1	–	–
SAR116_clade; alpha_proteobacterium	1	1	1	1	1	–	–
SAR116_clade; marine_metagenome	1	1	1	1	1	–	–
Rhodobacteraceae	1	1	1	1	1	–	–
Rhodobacteraceae; uncultured metagenome	1	1	1	1	1	–	–
Alphaproteobacteria; SAR11 Clade_Ia	1	1	1	1	1	–	–
Alteromona	1	1	1	1	1	–	–
Marinimicrobia; SAR406 clade	1	1	1	1	1	–	–
Flavobacteriaceae; NS2b marine group	1	1	1	1	1	–	–
SAR11_clade; Clade_Ia	1	1	1	1	1	–	–
Synechococcus_CC9902	1	1	1	1	1	–	–
**Seawater Indicator ASVs**							
Alphaproteobacteria; Rickettsiales; S25 593	0	0	1	1	1	0.0001	0.003
Pseudomonadales; SAR86_clade; uncultured SAR86	0	0	1	1	1	0.0001	0.003
SAR86_clade; marine_metagenome	0	0	1	1	1	0.0001	0.003
SAR11_clade_CladeIII; marine_metagenome	0	0	1	1	0.960	0.001	0.017
Pseudomonadales; OM182 clade	0	0	1	1	0.943	0.0011	0.017
SAR11; Clade I	0	0	1	1	0.913	0.002	0.029
SAR11; Clade III	0	0	1	1	0.972	0.0004	0.005
Alphaproteobacteria; SAR11 CladeI	0	0	1	1	0.943	0.0012	0.017
SAR92 clade	0	0	1	1	0.943	0.0013	0.018
Alphaproteobacteria; S25-593; uncultured_marine	0	0	1	1	0.913	0.003	0.033
Dadabacteriales; marine_metagenome	0	0	1	1	0.913	0.003	0.033
Dehalococcoidia; SAR202_clade	0	0	1	1	0.946	0.004	0.04
Porticoccaceae; SAR92_clade	0	0	1	1	0.912	0.006	0.04
Rhodospirillales; Magnetospiraceae	0	0	1	1	0.882	0.006	0.05
Myxococcales; Myxococcaceae P3OB 42	0	0	1	1	0.882	0.006	0.05

Supplementary Table 5: Results of indicator values (IndVal), for ASVs of Prokaryotes, highlighting both generalist ASVs found in Spring and Summer 2012 samples from bulk particles (PITs) and seawater (SW). The upper panel lists generalist ASVs consistently present across all tested sample groups, reflecting their broad distribution. The lower panel presents significant seawater indicator ASVs (FDR-adjusted p-value < 0.05; IndVal ≥ 0.8) for both seasons, specifically associated with seawater samples. Indicator values (IndVal), unadjusted p-values, and FDR-adjusted p-values are provided.

Generalist ASVs	PITS Spring	PITs Summer	SW Spring	SW Summer	IndVal	p–value	FDR p–value
Prochlorococcus_MIT9313_sp	1	1	1	1	1.00	–	–
Emiliania_huxleyi:plas	1	1	1	1	1.00	–	–
Synechococcus_CC9902_sp	1	1	1	1	1.00	–	–
Prymnesiophyceae_plas	1	1	1	1	1.00	–	–
Phaeocystis_sp:plas	1	1	1	1	0.957	–	–
Sarcinochrysidaceae_X_sp:plas	1	1	1	1	0.957	–	–
Prymnesiales:plas	1	1	1	1	0.935	–	–
Dictyochophyceae_XXX_sp:plas	1	1	1	1	0.935	–	–
Pelagomonas:plas	1	1	1	1	0.913	–	–
Chrysochromulina_camella:plas	1	1	1	1	0.866	–	–
**Seawater Indicator ASVs**							
Prochlorococcus_MIT9313_sp	0	0	1	1	0.979	0.0002	0.01

Supplementary Table 6: Results of Indicator Values for ASVs of photoautotroph (plastid and cyanobacteria), highlighting both generalist ASVs found in Spring and Summer 2012 samples from bulk particles (PITs) and seawater (SW). The upper panel lists generalist ASVs consistently present across all tested sample groups, reflecting their broad distribution. The lower panel presents significant seawater indicator ASVs (FDR-adjusted p-value < 0.05; IndVal ≥ 0.8) for both seasons, specifically associated with seawater samples. Indicator values (IndVal), unadjusted p-values, and FDR-adjusted p-values are provided. Note that the *Prochlorococcus*_MIT9313_sp listed among the generalist ASVs is a different ASV from the one listed in the seawater indicator ASVs.

II. Supplementary Text: *Enhancing Clustering Fidelity: A Methodological Perspective*

The Sargasso Sea has been extensively studied in terms of physicochemical parameters (Lomas et al., 2013; Steinberg et al., 2001), carbon export (Buesseler, 1998; Lomas et al., 2022), and the microbial communities (Amacher et al., 2009; Carlson et al., 2009; Ewart et al., 2008) found in this oligotrophic region, providing expectations on seasonal patterns in microbial composition and diversity. However, sampling efforts (Weiss et al., 2017) and natural community shifts (Bolaños et al., 2021) may obscure established ecological patterns, as observed in our samples. We amplified the 16S rRNA genes of bulk particle samples retrieved from Particle Interceptor Traps (PITs) impacted by mesoscale eddies. As mentioned in the **Main Text**, the microbial composition of the bulk particle community from samples collected in the cyclonic eddy C2 showed similarities with the surrounding seawater. Yet, one of the replicates (223) exhibited a higher contribution from a *Prochlorococcus* ASV compared to its counterpart (222), but both had a distinct contribution from ASVs with lower abundance (i.e., < 1%; 20 reads). While relative abundance analysis is a common approach in microbiology, it is important to recognize that shifts in relative abundance may not always reflect changes in absolute microbial community structure, as highlighted by Morton et al. (2019). Lin and Das Peddada (2020) emphasize that compositional data are inherently constrained and prone to spurious correlations, necessitating normalization techniques to accurately capture ecological dynamics. Without such adjustments, compositional shifts might reflect mathematical artifacts rather than true biological changes, particularly in datasets where dominant taxa skew diversity metrics or obscure rare but ecologically meaningful organisms. Therefore, this supplementary text aims to explicitly demonstrate the thought process of the data handling techniques applied to our samples.

Normalization techniques used in this study: To address the compositional biases inherent in microbiome datasets, we applied a log(x+1) transformation to the rarefied library, producing double-normalized data. These data were subsequently analyzed using Bray-Curtis dissimilarity and visualized through UPGMA dendrograms overlaid onto heatmaps. Gloor et al. (2017) note that microbiome datasets are frequently zero-inflated, making the addition of a pseudocount during log transformation a necessary step for stabilizing variance and ensuring comparability between low-abundance and high-abundance taxa. However, the same authors also acknowledge that the choice of pseudocount is inherently arbitrary and can influence results. Schloss (2024a) reinforces this point, arguing that pseudocounts should be carefully contextualized, as the lack of consensus on optimal pseudocount selection introduces variability into analyses. Despite these challenges, the combined use of rarefaction and log transformation in this study effectively addressed compositional biases and enhanced subtle ecological differences while preserving biologically meaningful patterns in microbial community data, as explained in the following sections. The following subsections elaborate on the rationale for the double normalization strategy adopted in this study, providing supporting literature, and evaluating the impacts of the adopted techniques to our analyses.

Rarefaction: McMurdie and Holmes (2014) argue that rarefaction unnecessarily discards data, increasing variance and potentially leading to both false positives and false negatives. As an alternative, Gloor et al. (2017) advocate for a “compositional approach” that bypasses rarefaction by applying log-ratio transformations (e.g., center-log-ratios) and compositional distance metrics (e.g., Aitchison) to normalize microbiome datasets and reduce compositional biases. However, Schloss (2024b) challenges the conclusions of McMurdie and Holmes (2014), reproducing their findings and identifying 11 methodological biases that skewed their study against rarefaction. Schloss argues that while rarefaction is often criticized as arbitrary, researchers can mitigate this by clearly defining a minimum library size criterion based on data quality. Additionally, Weiss et al. (2017) demonstrates the utility of rarefaction in cases of highly uneven sequencing depths (e.g., >10x differences in library size), showing that it minimizes bias from sampling effort and helps control false discovery rates (FDR). In the context of our data, which exhibited large differences in sequencing depth (**Supplementary Table 1**) rarefaction was important in reducing bias during the permutational tests implemented in this study such as Bray-Curtis-based NMDS ordination and UPGMA dendrogram, PERMANOVA and Indicator Species Analysis.

Permutational Tests and Differential Abundance Analysis on Rarefied Data: Rarefaction is often criticized for reducing statistical power in differential abundance testing, such as with DESeq2, as it can increase the likelihood of missing true positives. However, Hong et al. (2022) demonstrates that rarefaction can improve Type I error control, maintaining the error rate at ≤5% in datasets with highly uneven sequencing depths and pronounced overdispersion (i.e., when taxa abundances deviate from the Poisson distribution). As overdispersion increases, the rarefied libraries increasingly resemble the original data, preserving biologically relevant patterns when analyzed with DESeq2. This is attributed to DESeq2's reliance on the Negative Binomial (NB) model, which accounts for overdispersion more effectively than simpler distributional assumptions like the Poisson model. Additionally, Hong et al. (2022) highlights the robustness of rarefaction in permutational tests such as PERMANOVA, which was used in our study. Given our data's uneven sequencing depth (**Supplementary Table 1**) and overdispersion (**Supplementary Figure 12**), the combined use of rarefaction and DESeq2 enhanced the accuracy and biological relevance of our analyses.

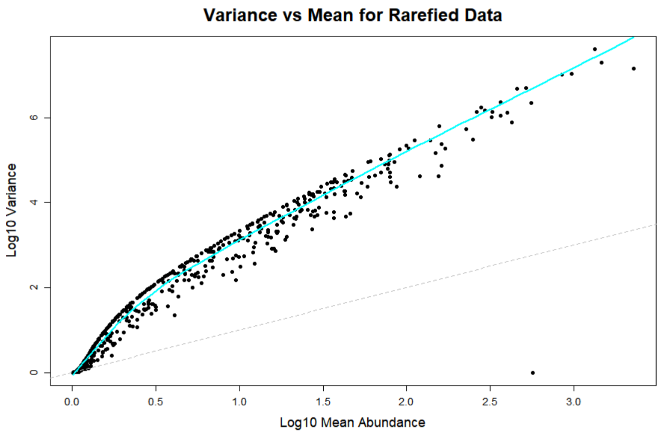

Supplementary Figure 12: Variance vs Mean for Rarefied Data of 16S rRNA gene amplicons of photoautotrophs. The x-axis represents the log10-transformed mean abundance of ASVs across all samples, while the y-axis represents the log10-transformed variance in ASV abundance. The dashed grey line shows the expected variance-to-mean relationship under a Poisson distribution, where variance equals the mean. The blue curve represents the fit to a Negative Binomial (NB) model, which accounts for overdispersion (variance exceeding the mean). The deviation of ASVs from the dashed line demonstrates that the data do not conform to the Poisson distribution assumption, instead exhibiting overdispersion.

Log-transforming rarefied data: The log(x+1) transformation of relative abundance in a rarefied table is redundant since rarefaction already standardizes library sizes to the same sequencing depth, which scales the library. However, log transformations address additional challenges that persist even after rarefaction, such as right-skewed distributions and zero inflation, which can obscure meaningful ecological patterns (McKnight et al., 2019). While rarefaction ensures comparability by equalizing sequencing depth across samples, it does not mitigate the disproportionate influence of highly abundant taxa on community analysis (Weiss et al., 2017). Log transformations stabilize variance and downweighs dominant taxa, allowing rare taxa to contribute more prominently to diversity measures and clustering patterns (Clarke et al., 2014). Gloor et al. (2017) emphasize the limitations of rarefaction and advocate for compositional approaches such as log-ratio transformations, but their broader argument supports the use of methods that address compositional biases and improve interpretability in microbiome datasets. In our context, applying a log(x+1) transformation to the rarefied table helped improve the resolution of subtle community differences by reducing the overwhelming influence of dominant ASVs, such as *Prochlorococcus*, thereby aligning our clustering results more closely with ecological expectations (explained in the following sections)

McMurdie and Holmes (2014) caution against the use of rarefaction, arguing that it leads to “the omission of available valid data,” potentially resulting in both loss of statistical power and distortion of ecological patterns. Schloss (2024b), however, highlights that rarefaction, while discarding data, can be less detrimental than the

filtering step often applied in compositional approaches. Filtering out low-prevalence sequences prior to normalization may inadvertently skew the community distribution and affect downstream diversity analyses. Similarly, the application of log transformations to rarefied data introduces its own set of challenges. McKnight et al. (2019), using simulated and natural gut microbiome datasets **without focusing on highly uneven sampling**, questioned the utility of applying log transformations to rarefied data. Their findings suggest that log transformations can artificially amplify differences among rare ASVs while suppressing variations in abundant ASVs, potentially distorting beta-diversity measures such as Bray-Curtis dissimilarity.

Comparison of normalization techniques and their effects on Bray-Curtis dissimilarity: To evaluate the impact of different normalization techniques on microbial community data, we compared Bray-Curtis dissimilarities calculated from the rarefied table (Actual BC) to those calculated from combinations of normalization techniques (Calculated BC). We assessed five normalization techniques against rarefaction using Person's correlation and root mean square error for 1) relative abundance, 2) percent relative abundance, 3) log-transformed relative abundance, 4) log-transformed percent relative abundance, and 5) log-transformed rarefied counts (**Supplementary Figure 13**).

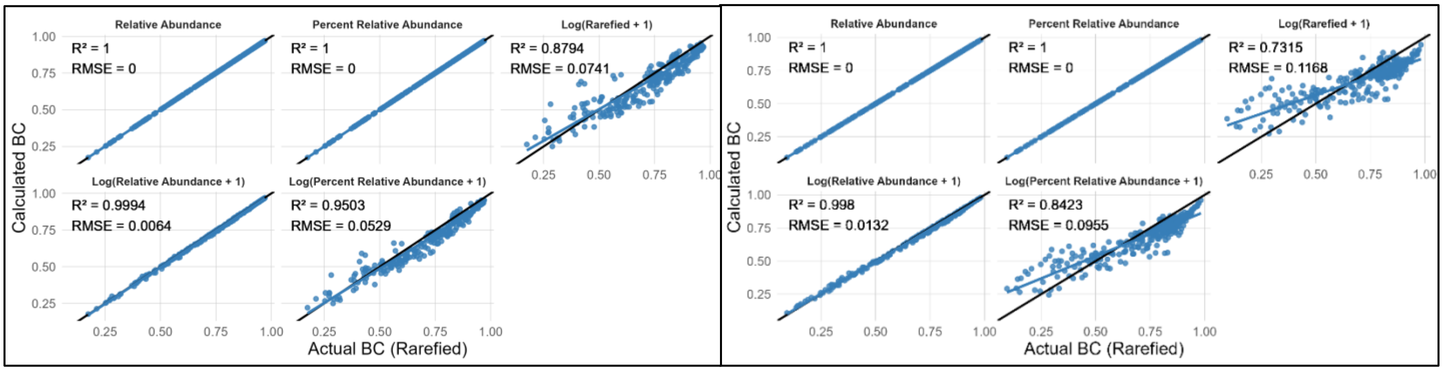

Supplementary Figure 13: Comparison of Bray-Curtis (BC) dissimilarities calculated using various normalization techniques for 16S rRNA gene data of prokaryotes (left panel) and photoautotrophs (right panel). Each panel shows the alignment between the Actual BC from the rarefied table (x-axis) and the Calculated BC for different transformations (y-axis). The black solid line represents perfect alignment (1:1), while the blue line shows the linear regression fit. R^2 values represent the strength of the linear relationship between the Bray-Curtis (BC) dissimilarities derived from the rarefied table and those calculated from the various normalization techniques. The root mean square error (RMSE) values indicate the average deviation between the BC dissimilarity values, interpreted as the average error (±) in BC dissimilarity introduced by each transformation.

For prokaryotic data, untransformed relative and percent relative abundance perfectly aligned with the Actual BC from the rarefied table (R^2 = 1, RMSE = 0). Log-transformed percent relative abundance (R^2 = 0.9503, RMSE = 0.0529) exhibited slightly better alignment with the rarefied table compared to log-transformed rarefied counts (R^2 = 0.8794, RMSE = 0.0741), indicating a moderate deviation. For plastids and cyanobacteria, untransformed relative and percent relative abundance had perfect alignment (R^2 = 1, RMSE = 0) similar to the prokaryotes, while log-transformed percent relative abundance showed higher similarity (R^2 = 0.8423, RMSE = 0.0955) compared to log-transformed rarefied counts (R^2 = 0.7315, RMSE = 0.1168). These values reflect greater variability and lower alignment in plastid and cyanobacteria data under log transformations compared to prokaryotic data. However, these findings demonstrate the contextual variability in the effectiveness of log transformations, as our results demonstrate a higher preservation of BC similarities post-transformation compared to the distortions reported by McKnight et al. (2019), possibly due to differences in dataset characteristics. The rest of the sections will focus on the plastid and cyanobacteria dataset discussion.

Comparisons of normalization techniques through dendrograms: The effects of different normalization techniques on clustering patterns were assessed using UPGMA dendrograms based on Bray-Curtis dissimilarity of photoautotrophs (**Supplementary Figure 14**). These dendrograms illustrate the hierarchical relationships

between samples, providing insights into how various normalization approaches influence clustering outcomes. This evaluation helps understand the impact of normalization methods on preserving ecological relationships while aiming to minimize biases introduced by dominant taxa. The six normalization techniques evaluated were: 1) rarefaction, 2) relative abundance, 3) percent relative abundance, 4) log-transformed relative abundance, 5) log-transformed percent relative abundance, and 6) log-transformed rarefied counts.

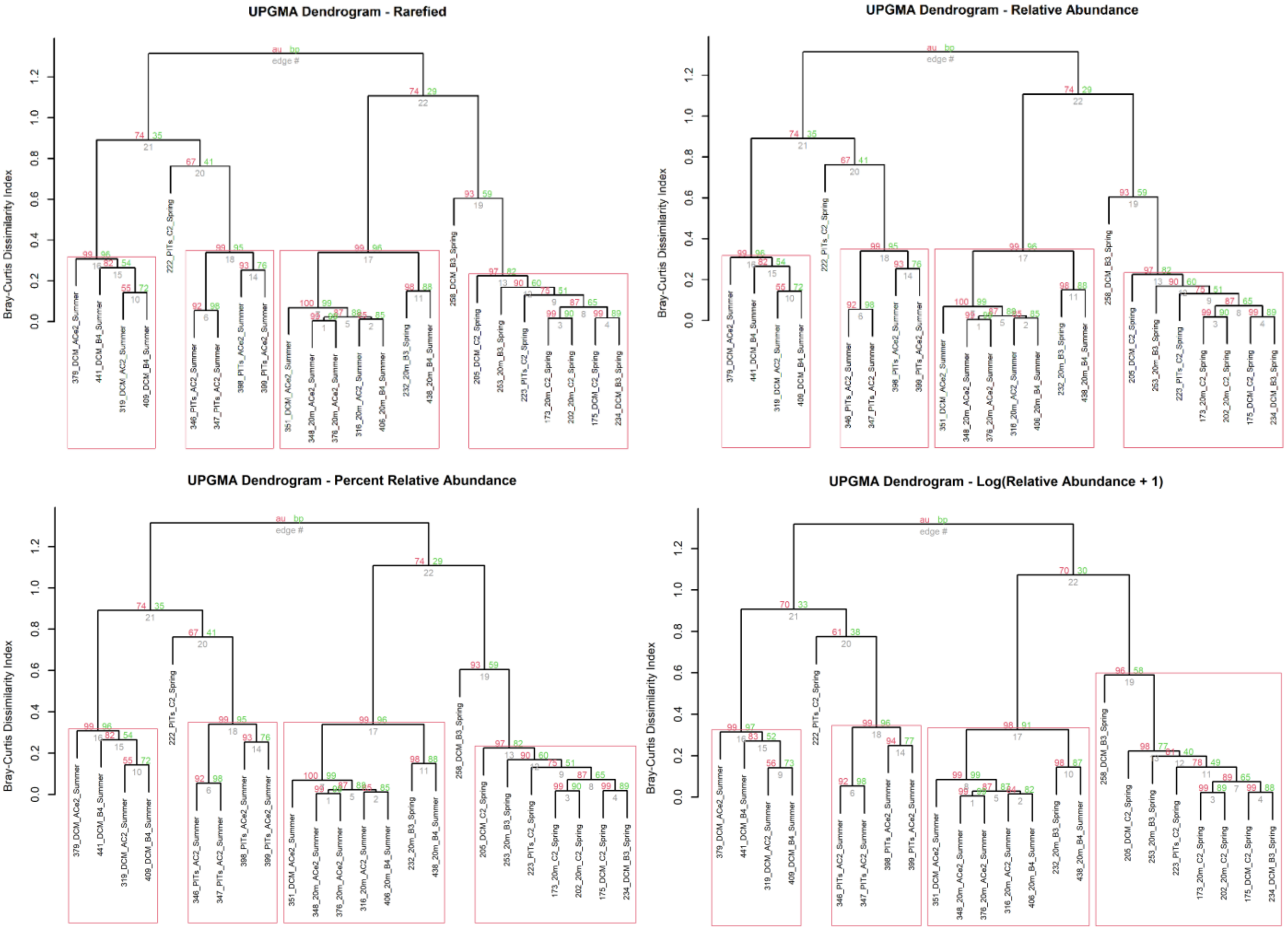

Supplementary Figure 14: UPGMA dendrograms based on Bray-Curtis dissimilarity indices, illustrating sample clustering patterns under different normalization techniques. Each dendrogram is derived from photoautotroph data normalized using one of the following methods: (top left) rarefied counts, (top right) relative abundance, (bottom left) percent relative abundance, and (bottom right) log(x+1)-transformed relative abundance. The red boxes highlight significant clusters with Approximately Unbiased (AU) p-values (in red, as percentages) above the 95% confidence threshold ($\alpha = 0.05$). Differences in clustering patterns reflect the influence of normalization techniques on sample relationships, with log transformations amplifying contributions from rare taxa while diminishing the dominance of highly abundant taxa.

The rarefied table produced clusters that closely aligned with known ecological groupings, reaffirming the utility of rarefaction for standardizing library sizes. However, notable discrepancies emerged, particularly among the bulk particle sample replicates from the cyclonic eddy C2, which did not cluster together. Dendrograms based on untransformed relative abundance and percent relative abundance produced identical clustering patterns to the rarefied table due to their similar scaling effects. In contrast, log-transformed techniques revealed distinct clustering patterns, especially among bulk particle samples from eddy C2. The log(x+1)-transformed rarefied table highlighted subtle ecological differences by amplifying the contributions of

less abundant taxa but reduced the dominance of highly abundant taxa, altering their influence on the overall clustering.

The first three dendrograms (rarefied table, relative abundance, and percent relative abundance; **Supplementary Figure 14**) exhibit identical branching and clustering patterns, as expected from the inherited scaling effect of rarefaction. However, the log(x+1) transformation (**Supplementary Figure 14**) shows notable changes in cluster membership, particularly for branch #19 and its subsequent leaves. This transformation removes the equal scaling achieved by rarefaction (sample no longer sums to the same value) by downweighing the contribution of dominant taxa, thereby altering clustering patterns. The results also show a decrease in the overall Approximately Unbiased (AU) p-values (interpreted as percentages with $\alpha = 5\%$ significance level) for clusters and branches that remained consistent with the untransformed dendrograms. These AU p-values will be further discussed in the next subsection.

Finally, **Supplementary Figure 15** shows log(x+1)-transformed rarefied table (left) and the log(x+1)-transformed percent relative abundance (right), where the clustering patterns for the bulk particle replicates from cyclonic eddy C2 (samples 222 and 223) converge. The red boxes highlight significant clusters with Approximately Unbiased (AU) p-values (in red, as percentages) above the 95% confidence threshold ($\alpha = 0.05$). Notably, the bulk particle replicates from the cyclonic eddy C2 (samples 222 and 223) cluster together in both dendrograms. However, the statistical support differs: the AU p-value for the 222/223 cluster is 100% in the log(x+1)-transformed rarefied table (right), whereas it is slightly lower at 94% in the log(x+1)-transformed percent relative abundance dendrogram (left). These subtle differences help understand the influence of normalization techniques on clustering fidelity and the statistical robustness of ecological interpretations.

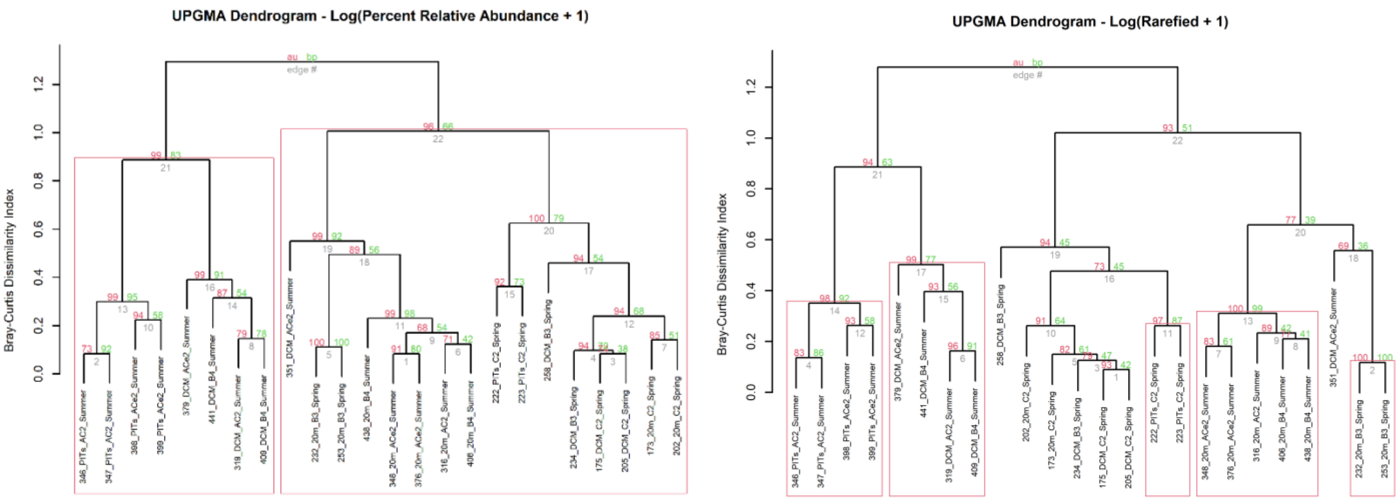

Supplementary Figure 15: UPGMA dendrograms based on Bray-Curtis dissimilarity indices, comparing sample clustering patterns under two normalization techniques: (left) log(x+1)-transformed percent relative abundance and (right) log(x+1)-transformed rarefied counts. The red boxes highlight significant clusters with Approximately Unbiased (AU) p-values (in red, as percentages) above the 95% confidence threshold ($\alpha = 0.05$). Differences in clustering patterns emphasize how each normalization technique impacts the relative influence of dominant and rare taxa on sample relationships.

Comparisons of Approximately Unbiased (AU) p-values and Adjusted Rand Index (ARI) Across Normalization Techniques: Building upon the observed clustering patterns, we evaluated the statistical confidence and alignment of clusters using Approximately Unbiased (AU) p-values and Adjusted Rand Index (ARI). While the highest AU p-value for the 222/223 replicate samples is observed in the log(x+1)-transformed rarefied table (100%), the log(x+1)-transformed percent relative abundance generally shows higher AU p-values across other

clusters. This suggests a slightly stronger overall statistical support for clustering patterns in the latter normalization approach.

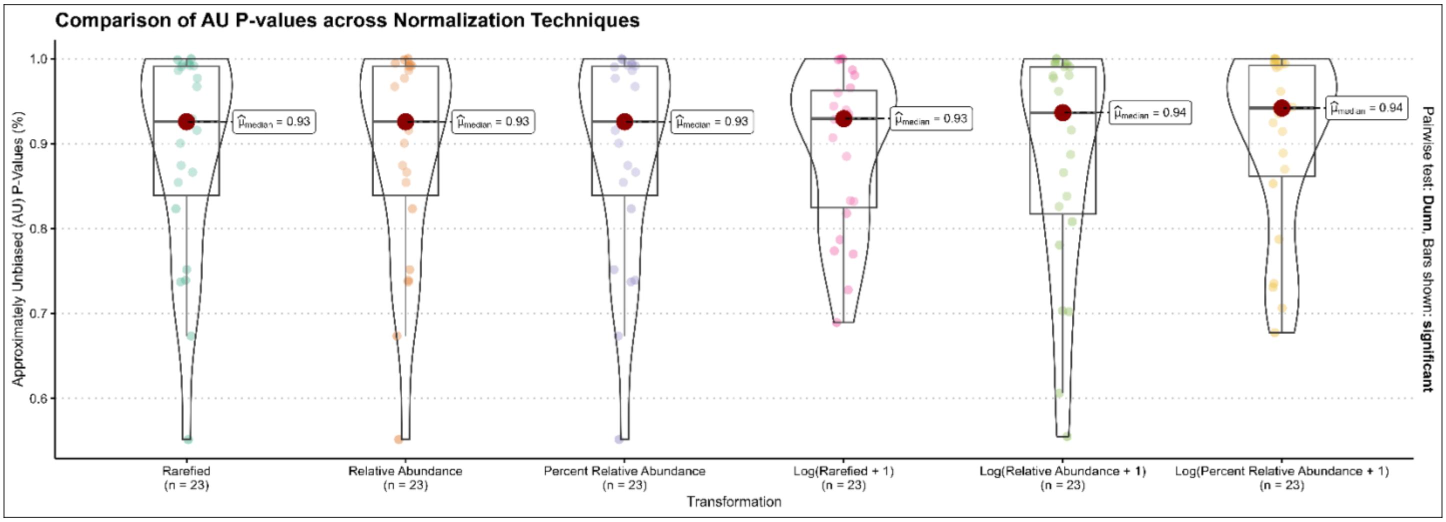

Supplementary Figure 16: Violin plots comparing Approximately Unbiased (AU) p-values across six normalization techniques: Rarefied, Relative Abundance, Percent Relative Abundance, and their log(x+1) transformations. Each plot represents the distribution of AU p-values for hierarchical clusters derived from UPGMA dendrograms based on Bray-Curtis dissimilarity. The red dot indicates the mean AU p-value for each normalization technique.

The violin plots (**Supplementary Figure 16**) highlight that no significant global differences exist between techniques, as evidenced by the absence of bars of significance. However, the distribution and range of AU p-values uncover key differences. The log(x+1)-transformed rarefied table and the log(x+1)-transformed percent relative abundance show narrower interquartile ranges, indicative of more consistent clustering confidence. The log(x+1)-transformed rarefied table displays a reduced upper quantile, suggesting fewer high-confidence clusters overall, while the log(x+1)-transformed percent relative abundance shows decreased variability in the lower quantile, reflecting stronger confidence in specific clusters, such as the 222/223 replicates.

In addition to AU p-values, the Adjusted Rand Index (ARI) comparison (**Supplementary Figure 17**) provides a complementary measure of clustering consistency relative to the rarefied table. ARI values quantify the similarity of clustering patterns across normalization techniques, with higher values indicating greater alignment with the rarefied table. For this analysis, we considered the dendrogram height—which reflects the BC index—and applied a cutoff of 0.9 to capture major, statistically supported clusters based on the BC dissimilarity index. Untransformed normalization techniques, such as relative abundance and percent relative abundance, show the highest ARI values, reflecting that these techniques preserve global clustering patterns and produce clusters identical to those from the rarefied table.

In contrast, log-transformed techniques exhibit progressively lower ARI values, indicating deviations in cluster memberships. Specifically, the log(x+1)-transformed relative abundance introduces the least shifts, whereas the log(x+1)-transformed percent relative abundance and the log(x+1)-transformed rarefied table display the same divergence at or above 0.9 tree height. These deviations highlight the trade-offs in log transformations: while they enhance the resolution of subtle ecological differences by emphasizing contributions from less abundant taxa, they also change global clustering patterns. Whether these changes represent an improvement depends on the ecological context and the specific research question, as log-transformed techniques may better capture nuanced biological variation in certain scenarios while potentially obscuring broader clustering trends (Feng et al., 2014; Packard, 2014).

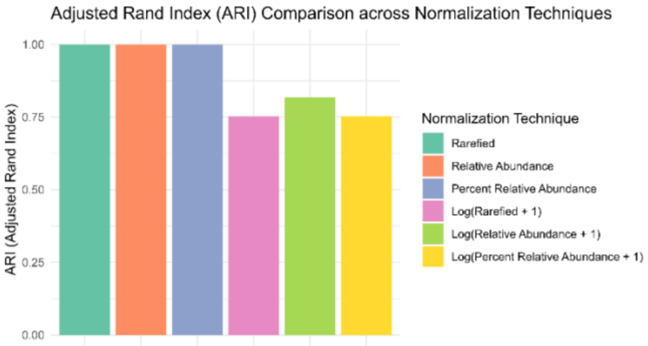

Supplementary Figure 17: Bar plot showing Adjusted Rand Index (ARI) comparisons across six normalization techniques: Rarefied, Relative Abundance, Percent Relative Abundance, and their log(x+1) transformations. ARI values represent the similarity of clustering patterns derived from each normalization technique relative to the rarefied data, with higher values indicating closer alignment.

Effects of Log(x+1) transformations across normalization techniques: The log(x+1) transformation is a widely used method for balancing contributions from dominant and rare taxa (e.g., Cruz et al., 2021; Robertson et al., 2013). This transformation reduces the disproportionate influence of highly abundant groups (e.g., *Synechococcus* and *Prochlorococcus* in our study), while enhancing the visibility of less abundant taxa (Clarke et al., 2014). However, its impact depends heavily on the starting normalization technique and the range of the data, influencing clustering patterns and interpretability:

- **Log(x+1) Transformation of Relative Abundance (Supplementary Figure 18):** This transformation removes the proportional scale inherent to rarefaction that standardizes taxa contributions across samples, which was preserved during relative abundance calculations. Despite the transformation, the original dominant taxa continue to dominate proportions, rendering the transformation redundant to rarefaction. As shown previously, it does not offer significant statistical or clustering changes over the untransformed relative abundance.

- **Log(x+1) Transformation of Rarefied Counts (Supplementary Figure 19):** This approach improves clustering by reducing the dominant effect of highly abundant taxa, allowing less dominant taxa to contribute more to Bray-Curtis dissimilarity. However, the broad range of the rarefied data (e.g., 0–1,000s) compared to the ranges of other normalization techniques, amplifies the downweighing effect, which can obscure the contributions of dominant taxa like Rhizosolenia.

- **Log(x+1) Transformation of Percent Relative Abundance (Supplementary Figure 20):** This normalization combination strikes a balance between the two approaches above. The shorter range of percent relative abundance (e.g., 0–100%) compared to rarefied counts limits the extent of downweighing.

Thus, the choice of log transformation must consider its impact on the scale of the data and the specific goals of the analysis. Heatmaps overlaid with UPGMA dendrograms (**Supplementary Figures 18–20**) further illustrate the differing impacts of these transformations on clustering and visualization.

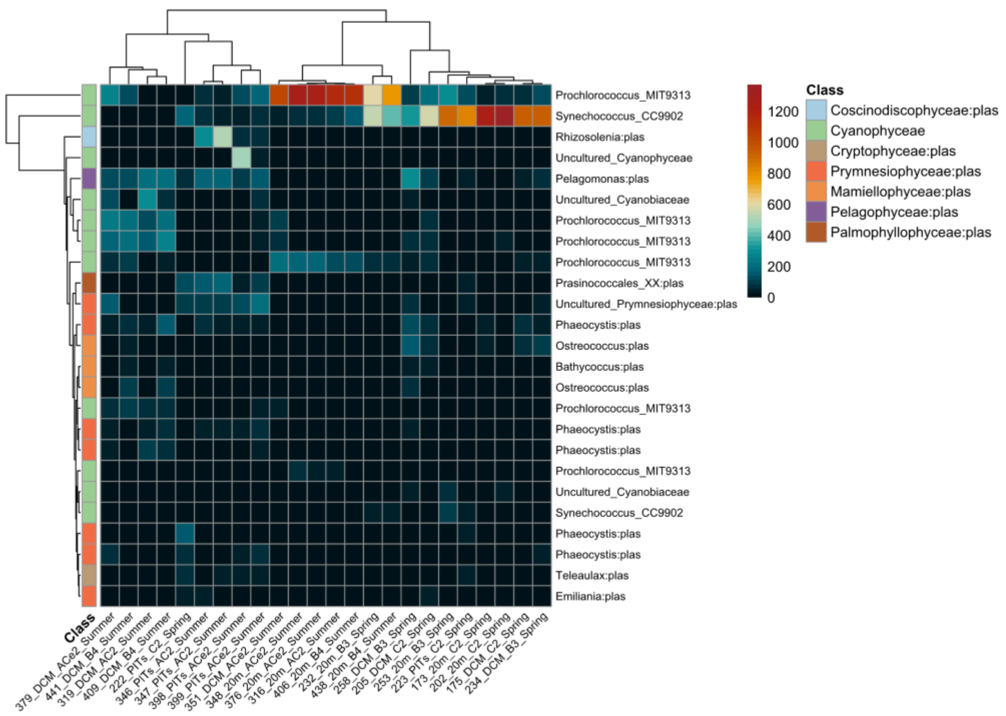

Supplementary Figure 18: Heatmap displaying the top 25 ASVs, overlaid with Bray-Curtis-based UPGMA dendrograms. The top dendrogram illustrates sample clustering patterns, while the left dendrogram highlights ASV abundance variations. Both dendrograms and the heatmap are derived from the rarefied table. The color gradient represents ASV abundance, with taxa annotated at the genus level and color-coded by class. The top dendrogram corresponds to **Supplementary Figure 15**.

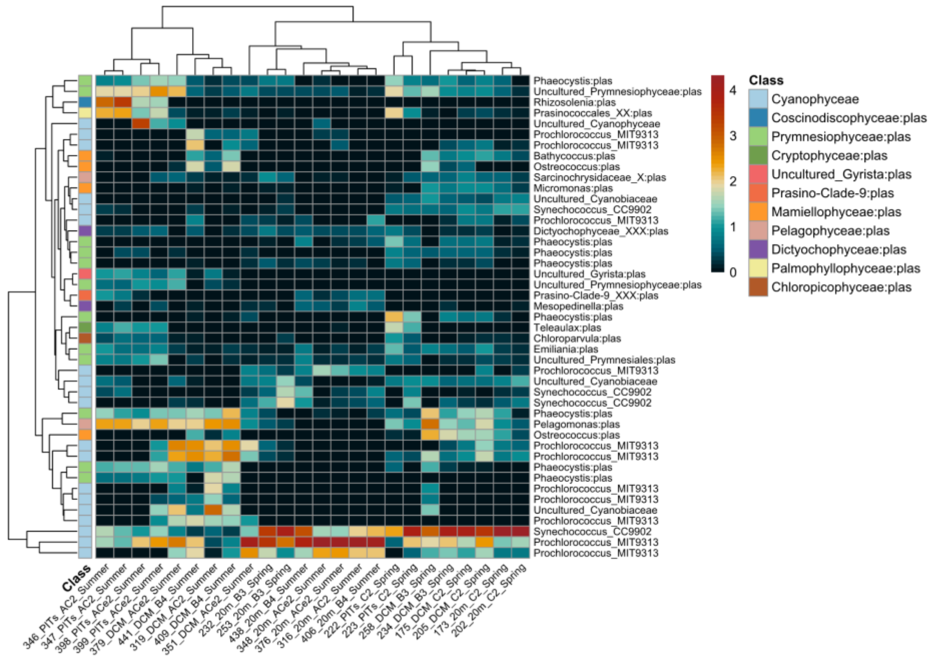

Supplementary Figure 119: Heatmap displaying the top 45 ASVs, overlaid with Bray-Curtis-based UPGMA dendrograms. The top dendrogram illustrates sample clustering patterns, while the left dendrogram highlights ASV abundance variations. Both dendrograms and the heatmap are derived from the log(x+1)-transformed percent relative abundance of the rarefied table. The color gradient represents ASV abundance, with taxa annotated at the genus level and color-coded by class. The top dendrogram corresponds to **Supplementary Figure 14**.

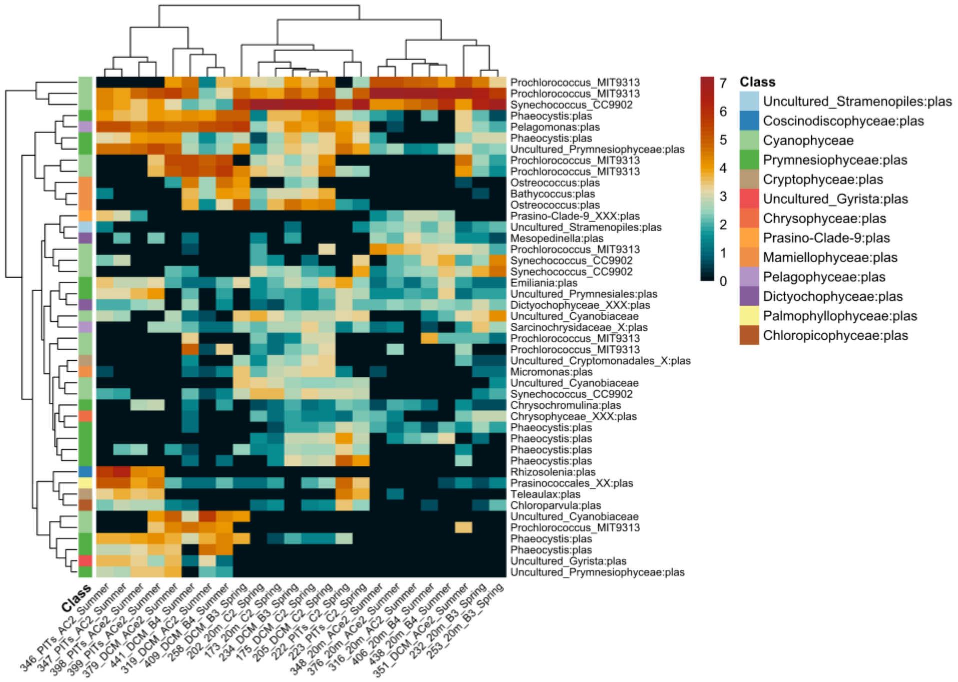

Supplementary Figure 20: Heatmap displaying the top 45 ASVs, overlaid with Bray-Curtis-based UPGMA dendrograms. The top dendrogram illustrates sample clustering patterns, while the left dendrogram highlights ASV abundance variations. Both dendrograms and the heatmap are derived from the log(x+1)-transformed rarefied table. The color gradient represents ASV abundance, with taxa annotated at the genus level and color-coded by class. The top dendrogram corresponds to **Supplementary Figure 14**.

The overrepresentation of *Rhizosolenia* (focal ASV corroborated through relative abundance, indicator species analysis and differential abundance analysis) on the trap material from the summer anticyclonic eddy was apparent in the three heatmaps. However, the log(x+1) rarefied table necessitated the inclusion of 45 ASVs for *Rhizosolenia* to appear in the list, unlike the heatmaps based on the rarefied table or on the log(x+1)-transformed percent relative abundance. This is because the downweighing effect of the log(x+1) transformation obscured the contribution of *Rhizosolenia* relative to less abundant taxa. As mentioned above, these differences in data behavior are due to the range of the data—a mixture of low and high values in a constrained range (i.e., 0–100%) helps with visualization and clustering purposes when log transformed. However, when combining the linear regression analysis on Actual BC vs Calculated BC (**Supplementary Figure 13**) with the UPGMA dendrograms (**Supplementary Figures 14–15**) and their Approximately Unbiased p-values analysis (**Supplementary Figures 16–17**), the log(x+1) transformation of **the rarefied table strikes as the most appropriate approach for our data**, as explain in the following section.

Ecological meaning to clustering patterns and concluding remarks: In our study, we demonstrate that particles collected across both seasons harbor a "core" microbiome (**Main Text Figures 2 & 6**) despite difference in environmental conditions. Clustering patterns of photoautotrophs assessed through NMDS plots (**Supplementary Figures 10**) align with *a priori* groupings—namely, water column versus bulk particle samples—with a 95% confidence level, despite some overlap in ellipses. PERMANOVA analysis on photoautotrophs (**Supplementary Table 4**) revealed that sample type (seawater versus bulk particle samples) explains 67.5% (FDR-corrected p-value = 0.001) of the community variation observed in Bray-Curtis dissimilarity of the rarefied table across both seasons. However, this distinction was less pronounced during the spring season, explaining only 36.7% of the variation (FDR-corrected p-value = 0.001). This reduction likely reflects differences among particle samples as well as seasonal influences on community composition.

Clustering patterns from the UPGMA dendrogram (**Supplementary Figure 14**) and NMDS plots (**Supplementary Figure 10**) based on the rarefied table of photoautotrophs suggest that the particle sample 223

from the spring C2 eddy resembles a water column community more than other particle samples. Specifically, sample 222 did not cluster with any group, while sample 223 clustered with spring water column samples, albeit with a relatively low Approximately Unbiased (AU) p-value of 75%. To further investigate these patterns, we assessed the impact of different normalization techniques on Bray-Curtis dissimilarity measures, aiming to mitigate the influence of overrepresented taxa, such as cyanobacteria, in bulk particle samples—particularly within the spring cyclone trap replicates—and to reveal meaningful ecological patterns.

The normalization techniques combined in this study for the dendrogram and heatmap helped resolve the ecological patterns in our samples, especially considering seasonal variability, eddy impact and the challenges posed by uneven sequencing depths. For instance, while summer particles exhibited diatom dominance and clustered distinctly from spring particles, spring particles collected from the cyclonic eddy C2 had difficulty clustering together. This was likely influenced by proportional differences in picocyanobacterial ASVs, yet subtle variations in lower-abundance taxa compared to surrounding water column samples suggested underlying similarities between the particles. Using Bray-Curtis dissimilarity with the log(x+1)-transformed rarefied data minimized the influence of dominant taxa, enhancing the clustering of replicates 222 and 223 from the spring eddy C2. This normalization strategy preserved the summer PITs and DCM deep-branch clusters in the UPGMA dendrogram derived from the rarefied table while improving the resolution of subtle community distinctions in the summer 20 m communities and spring samples. The regression analysis of this transformation ($R^2 = 0.7315$; **Supplementary Figure 13**), while presenting the highest effect, it reflects a moderate alignment between clustering patterns derived from the log(x+1)-transformed rarefied table and the original rarefied table, demonstrating the method's ability to retain much of the original structure while introducing meaningful changes in Bray-Curtis dissimilarities. By downweighing dominant taxa and amplifying the contributions of less abundant taxa, this approach strikes a balance between maintaining clustering fidelity and uncovering previously obscured ecological distinctions.

IV. Supplementary References

Amacher, J., Neuer, S., Anderson, I., & Massana, R. (2009). Molecular approach to determine contributions of the protist community to particle flux. *Deep Sea Research Part I: Oceanographic Research Papers*, *56*(12), 2206–2215. https://doi.org/10.1016/j.dsr.2009.08.007

Bolaños, L. M., Choi, C. J., Worden, A. Z., Baetge, N., Carlson, C. A., & Giovannoni, S. (2021). Seasonality of the Microbial Community Composition in the North Atlantic. *Frontiers in Marine Science*, *8*, 624164. https://doi.org/10.3389/fmars.2021.624164

Buesseler, K. O. (1998). The decoupling of production and particulate export in the surface ocean. *Global Biogeochemical Cycles*, *12*(2), 297–310. https://doi.org/10.1029/97GB03366

Buesseler, K. O., Antia, A. N., Chen, M., Fowler, S. W., Gardner, W. D., Gustafsson, O., Harada, K., Michaels, A. F., van der Loeff, M. R., Sarin, M., Steinberg, D. K., & Trull, T. (2007). An assessment of the use of sediment traps for estimating upper ocean particle fluxes. *Journal of Marine Research*, *65*(3), 345–416. https://doi.org/10.1357/002224007781567621

Callahan, B. J., McMurdie, P. J., Rosen, M. J., Han, A. W., Johnson, A. J. A., & Holmes, S. P. (2016). DADA2: High-resolution sample inference from Illumina amplicon data. *Nature Methods*, *13*(7), 581–583. https://doi.org/10.1038/nmeth.3869

Carlson, C. A., Morris, R., Parsons, R., Treusch, A. H., Giovannoni, S. J., & Vergin, K. (2009). Seasonal dynamics of SAR11 populations in the euphotic and mesopelagic zones of the northwestern Sargasso Sea. *The ISME Journal*, *3*(3), 283–295. https://doi.org/10.1038/ismej.2008.117

Cianca, A., Godoy, J. M., Martin, J. M., Perez-Marrero, J., Rueda, M. J., Llinás, O., & Neuer, S. (2012). Interannual variability of chlorophyll and the influence of low-frequency climate modes in the North Atlantic subtropical gyre. *Global Biogeochemical Cycles*, *26*(2). https://doi.org/10.1029/2010GB004022

Clarke et al., 2014-Change in Marine Communities An Approach to Statistical Analysis.pdf. (n.d.).

Cotti-Rausch, B. E., Lomas, M. W., Lachenmyer, E. M., Goldman, E. A., Bell, D. W., Goldberg, S. R., & Richardson, T. L. (2016). Mesoscale and sub-mesoscale variability in phytoplankton community

composition in the Sargasso Sea. *Deep-Sea Research Part I: Oceanographic Research Papers*, *110*, 106–122. https://doi.org/10.1016/j.dsr.2015.11.008

Cruz, B. N., Brozak, S., & Neuer, S. (2021). Microscopy and DNA -based characterization of sinking particles at the Bermuda Atlantic Time-series Study station point to zooplankton mediation of particle flux. *Limnology and Oceanography*, *66*(10), 3697–3713. https://doi.org/10.1002/lno.11910

De Cáceres, M., Legendre, P., & Moretti, M. (2010). Improving indicator species analysis by combining groups of sites. *Oikos*, *119*(10), 1674–1684. https://doi.org/10.1111/j.1600-0706.2010.18334.x

De Martini, F., Neuer, S., Hamill, D., Robidart, J., & Lomas, M. W. (2018). Clade and strain specific contributions of Synechococcus and Prochlorococcus to carbon export in the Sargasso Sea. *Limnology and Oceanography*, *63*(2007), S448–S457. https://doi.org/10.1002/lno.10765

Decelle, J., Romac, S., Stern, R. F., Bendif, E. M., Zingone, A., Audic, S., Guiry, M. D., Guillou, L., Tessier, D., Le Gall, F., Gourvil, P., Dos Santos, A. L., Probert, I., Vaulot, D., de Vargas, C., & Christen, R. (2015). Phyto REF: A reference database of the plastidial 16S rRNA gene of photosynthetic eukaryotes with curated taxonomy. *Molecular Ecology Resources*, *15*(6), 1435–1445. https://doi.org/10.1111/1755-0998.12401

Ewart, C. S., Meyers, M. K., Wallner, E. R., McGillicuddy, D. J., & Carlson, C. A. (2008). Microbial dynamics in cyclonic and anticyclonic mode-water eddies in the northwestern Sargasso Sea. *Deep Sea Research Part II: Topical Studies in Oceanography*, *55*(10–13), 1334–1347. https://doi.org/10.1016/j.dsr2.2008.02.013

Feng, C., Wang, H., Lu, N., Chen, T., He, H., Lu, Y., & Tu, X. M. (2014). *Log-transformation and its implications for data analysis*. *26*(2).

Gloor, G. B., Macklaim, J. M., Pawlowsky-Glahn, V., & Egozcue, J. J. (2017). Microbiome Datasets Are Compositional: And This Is Not Optional. *Frontiers in Microbiology*, *8*, 2224. https://doi.org/10.3389/fmicb.2017.02224

Guillou, L., Bachar, D., Audic, S., Bass, D., Berney, C., Bittner, L., Boutte, C., Burgaud, G., De Vargas, C., Decelle, J., Del Campo, J., Dolan, J. R., Dunthorn, M., Edvardsen, B., Holzmann, M., Kooistra, W. H.

C. F., Lara, E., Le Bescot, N., Logares, R., … Christen, R. (2013). The Protist Ribosomal Reference database (PR2): A catalog of unicellular eukaryote Small Sub-Unit rRNA sequences with curated taxonomy. *Nucleic Acids Research*, *41*(D1), 597–604. https://doi.org/10.1093/nar/gks1160

Hong, J., Karaoz, U., De Valpine, P., & Fithian, W. (2022). To rarefy or not to rarefy: Robustness and efficiency trade-offs of rarefying microbiome data. *Bioinformatics*, *38*(9), 2389–2396. https://doi.org/10.1093/bioinformatics/btac127

Lin, H., & Peddada, S. D. (2020). Analysis of microbial compositions: A review of normalization and differential abundance analysis. *Npj Biofilms and Microbiomes*, *6*(1), 60. https://doi.org/10.1038/s41522-020-00160-w

Lomas, M. W., Bates, N. R., Johnson, R. J., Knap, A. H., Steinberg, D. K., & Carlson, C. A. (2013). Two decades and counting: 24-years of sustained open ocean biogeochemical measurements in the Sargasso Sea. *Deep Sea Research Part II: Topical Studies in Oceanography*, *93*, 16–32. https://doi.org/10.1016/j.dsr2.2013.01.008

Lomas, M. W., Bates, N. R., Johnson, R. J., Steinberg, D. K., & Tanioka, T. (2022). Adaptive carbon export response to warming in the Sargasso Sea. *Nature Communications*, *13*(1), 1211. https://doi.org/10.1038/s41467-022-28842-3

McGillicuddy, D. J., Kosnyrev, V. K., Ryan, J. P., & Yoder, J. A. (2001). Covariation of mesoscale ocean color and sea-surface temperature patterns in the Sargasso Sea. *Deep-Sea Research Part II: Topical Studies in Oceanography*, *48*(8–9), 1823–1836. https://doi.org/10.1016/S0967-0645(00)00164-8

McKnight, D. T., Huerlimann, R., Bower, D. S., Schwarzkopf, L., Alford, R. A., & Zenger, K. R. (2019). Methods for normalizing microbiome data: An ecological perspective. *Methods in Ecology and Evolution*, *10*(3), 389–400. https://doi.org/10.1111/2041-210X.13115

McMURDIE, P. J., & Holmes, S. (2011). phyloseq: A Bioconductor Package For Handling And Analysis Of High-Throughput Phylogenetic Sequence Data. *Biocomputing 2012*, 235–246. https://doi.org/10.1142/9789814366496_0023

McMurdie, P. J., & Holmes, S. (2014). Waste Not, Want Not: Why Rarefying Microbiome Data Is Inadmissible. *PLoS Computational Biology*, *10*(4), e1003531. https://doi.org/10.1371/journal.pcbi.1003531

Morton, J. T., Marotz, C., Washburne, A., Silverman, J., Zaramela, L. S., Edlund, A., Zengler, K., & Knight, R. (2019). Establishing microbial composition measurement standards with reference frames. *Nature Communications*, *10*(1), 2719. https://doi.org/10.1038/s41467-019-10656-5

Nelson, N. B., Siegel, D. A., & Yoder, J. A. (2004). The spring bloom in the northwestern Sargasso Sea: Spatial extent and relationship with winter mixing. *Deep-Sea Research Part II: Topical Studies in Oceanography*, *51*(10-11 SPEC. ISS.), 987–1000. https://doi.org/10.1016/j.dsr2.2004.02.001

Packard, G. C. (2014). On the use of log-transformation versus nonlinear regression for analyzing biological power laws: Analyzing Biological Power Laws. *Biological Journal of the Linnean Society*, *113*(4), 1167–1178. https://doi.org/10.1111/bij.12396

Quast, C., Pruesse, E., Yilmaz, P., Gerken, J., Schweer, T., Yarza, P., Peplies, J., & Glöckner, F. O. (2013). The SILVA ribosomal RNA gene database project: Improved data processing and web-based tools. *Nucleic Acids Research*, *41*(D1), 590–596. https://doi.org/10.1093/nar/gks1219

Robertson, O. J., McAlpine, C., House, A., & Maron, M. (2013). Influence of Interspecific Competition and Landscape Structure on Spatial Homogenization of Avian Assemblages. *PLoS ONE*, *8*(5), e65299. https://doi.org/10.1371/journal.pone.0065299

Robeson, M. S., O'Rourke, D. R., Kaehler, B. D., Ziemski, M., Dillon, M. R., Foster, J. T., & Bokulich, N. A. (2021). RESCRIPt: Reproducible sequence taxonomy reference database management. In *PLoS Computational Biology* (Vol. 17, Issue 11). https://doi.org/10.1371/journal.pcbi.1009581

Schloss, P. D. (2024a). Rarefaction is currently the best approach to control for uneven sequencing effort in amplicon sequence analyses. *mSphere*, *9*(2), e00354-23. https://doi.org/10.1128/msphere.00354-23

Schloss, P. D. (2024b). Waste not, want not: Revisiting the analysis that called into question the practice of rarefaction. *mSphere*, *9*(1), e00355-23. https://doi.org/10.1128/msphere.00355-23

Severns, P. M., & Sykes, E. M. (2020). Indicator Species Analysis: A Useful Tool for Plant Disease Studies. *Phytopathology®*, *110*(12), 1860–1862. https://doi.org/10.1094/PHYTO-12-19-0462-LE

Siegel, D. A. (1990). Meridional variations of the springtime phytoplankton community in the Sargasso Sea. *Journal of Marine Research*, *48*(2), 379–412. https://doi.org/10.1357/002224090784988791

Steinberg, D. K., Carlson, C. A., Bates, N. R., Johnson, R. J., Michaels, A. F., & Knap, A. H. (2001). Overview of the US JGOFS Bermuda Atlantic Time-series Study (BATS): A decade-scale look at ocean biology and biogeochemistry. *Deep Sea Research Part II: Topical Studies in Oceanography*, *48*(8–9), 1405–1447. https://doi.org/10.1016/S0967-0645(00)00148-X

Sweeney, E. N., McGillicuddy, D. J., & Buesseler, K. O. (2003). Biogeochemical impacts due to mesoscale eddy activity in the Sargasso Sea as measured at the Bermuda Atlantic Time-series Study (BATS). *Deep-Sea Research Part II: Topical Studies in Oceanography*, *50*(22–26), 3017–3039. https://doi.org/10.1016/j.dsr2.2003.07.008

Weiss, S., Xu, Z. Z., Peddada, S., Amir, A., Bittinger, K., Gonzalez, A., Lozupone, C., Zaneveld, J. R., Vázquez-Baeza, Y., Birmingham, A., Hyde, E. R., & Knight, R. (2017). Normalization and microbial differential abundance strategies depend upon data characteristics. *Microbiome*, *5*(1), 27. https://doi.org/10.1186/s40168-017-0237-y
